# Actual sexual risk and perceived risk of HIV acquisition among HIV-negative men who have sex with men in Toronto, Canada

**DOI:** 10.1186/s12889-016-2859-6

**Published:** 2016-03-11

**Authors:** Maya A. Kesler, Rupert Kaul, Juan Liu, Mona Loutfy, Dionne Gesink, Ted Myers, Robert S. Remis

**Affiliations:** Dalla Lana School of Public Health, University of Toronto, Toronto, ON Canada; Department of Medicine, University Health Network, University of Toronto, Toronto, ON Canada; Women’s College Research Institute, Women’s College Hospital, University of Toronto, Toronto, ON Canada; Maple Leaf Medical Clinic, Toronto, ON Canada

**Keywords:** Human immunodeficiency virus, Men who have sex with men, Perceived risk, Actual risk, Principal components analysis, Toronto, Canada

## Abstract

**Background:**

Theory suggests that perceived human immunodeficiency virus (HIV) risk and actual HIV risk behaviour are cyclical whereby engaging in high risk behaviour can increase perceived risk, which initiates precautionary behaviour that reduces actual risk, and with time reduces perceived risk. While current perceived risk may impact future actual risk, it is less clear how previous actual risk shapes current perceived risk. If individuals do not base their current perceived risk on past behaviour, they lose the protective effect of perceived risk motivating precautionary behaviour. Our goal was to determine the impact of actual risk on perceived risk.

**Methods:**

Sexually active men who have sex with men (MSM) were recruited at the Maple Leaf Medical Clinic in downtown Toronto from September 2010 to June 2012. Participants completed a socio-behavioural questionnaire using an Audio Computer Assisted Self-Interview (ACASI). Actual HIV risk (primary predictor) was constructed by applying principal component analysis (PCA) to eight sexual risk survey questions and comprised three components which reflected sex with casual partners, sex with HIV-positive regular partners and sex with HIV unknown status regular partners. Perceived HIV risk (outcome) was measured by asking participants what the chances were that they would ever get HIV. Multivariable logistic regression was used to measure the association between actual and perceived HIV risk.

**Results:**

One hundred and fifty HIV-negative MSM were recruited (median age 44.5 years [IQR 37–50 years]). Twenty percent of MSM perceived their HIV risk to be high. The odds of having a high perceived risk was significantly higher in those with high actual HIV risk indicated by low condom use with an HIV-positive regular partner compared to those with low actual HIV risk indicated by high condom use with an HIV-positive regular partner (Odds Ratio (OR) 18.33, 95 % confidence interval (CI) 1.65–203.45). Older age was associated with lower perceived risk but only age 40–49 compared to less than 30 was statistically significant (OR 0.12, 95 % CI 0.016–0.86). The odds of having high perceived risk was significantly associated with men who used poppers in the previous 6 months compared to those who did not use poppers (OR 5.64, 95 % CI 1.20–26.48).

**Conclusions:**

Perceived HIV risk increased significantly as condom use with an HIV-positive regular partner decreased. However, perceived HIV risk was not associated with condom use with casual partners or HIV unknown status regular partners, even though these behaviours could be considered risky. The relationship between perceived and actual risk in HIV studies is complex and has implications on how health care workers address the issue of risky sexual behaviour and perceived risk.

**Electronic supplementary material:**

The online version of this article (doi:10.1186/s12889-016-2859-6) contains supplementary material, which is available to authorized users.

## Background

In Canada in 2014, an estimated 75,500 people were living with HIV/AIDS and men who have sex with men (MSM) were disproportionately affected making up 49 % of prevalent infections, and 54% of incident infections [1]. In Ontario in 2012, there were 458 HIV diagnoses among MSM, accounting for 48.5 % of all new HIV diagnoses [2]. Of those cases, almost two-thirds were diagnosed in Toronto, the largest metropolitan area in the province of Ontario [2]. Among MSM in 2009, Toronto had the highest annual HIV incidence rate of all Ontario health regions at 1.0 % [3]. Factors that affect the risk of HIV acquisition in MSM include type of sexual act, contact with ejaculate or bodily fluids, presence of other sexually transmitted infections (STIs), the viral load of their HIV-infected sexual partner, their circumcision status, and drug use [4–9]. Condomless receptive anal intercourse with an HIV-positive partner has been found to be the most important sexual risk factor for HIV transmission and acquisition [6].

The relationship between perceived and actual risk is complex and is a central component of many protective health behaviour models [10]. These theories hypothesize that engaging in high risk behaviour can increase perceived risk, which can then motivate the avoidance of risky behaviour [10]. When that behaviour is avoided, perceived risk then decreases [10]. For this reason, the relationship between the two constructs is seen as reciprocal [10, 11]. Perceived risk has been found to be significantly but only weakly associated with risky behaviour [10, 12–14]. This suggests that people are appropriately accounting for their sexual behaviour when reporting their perceived vulnerability to HIV [15, 16]. However, this is not a constant relationship since many individuals perceive themselves to be at little or no risk for HIV while simultaneously engaging in risky sexual behaviour [17–19]. In this context, denial or distancing of risk may be at play, as a mechanism to reduce the fear of infection [20]. Adopting this rationalization may lead to the belief that engaging in certain behaviours is not risky when it actually is [20]. One concern is that if high risk persons do not acknowledge their current perceived risk based on their past behaviour, they lose the protective effect of perceived risk to motivate precautionary behaviour [10].

One reason why the relationship between perceived and actual risk is inconsistent is because there is no perfect construction of an actual risk variable. There are a range of sexual and behavioural variables that could be incorporated into the definition of actual risk, but there is no firm consensus among researchers. The HIV Incidence Risk Index for MSM (HIRI-MSM) is one index that attempts to quantify actual HIV risk [21]. It is highly regarded because it enables physicians to quickly and accurately assess a person’s risk level. However, it incorporates drug use and age variables that may not be appropriate in all cases and requires specific scales which may not be available to researchers. Therefore, a method that takes into account all sexual risk variables, but reduces the number of variables required in a model, and accounts for the most variance is ideal. This method is Principal Components Analysis (PCA) [22].

The purpose of this study was to examine the association between MSM’s self-reported current perceived risk of HIV infection in light of their self-reported sexual behaviour in the previous 6 months and their actual HIV risk, as constructed using PCA.

## Methods

### Study setting and eligibility

This secondary data analysis uses data derived from HIV-negative men enrolled in the Toronto HIV/STI Co-Infections Study. The overarching purpose of the STI/HIV Co-Infections Study was to elucidate how STIs enhance HIV susceptibility and secondary transmission [23]. HIV-positive and HIV-negative MSM were recruited through the Maple Leaf Medical Clinic (MLMC), a large primary care and HIV-related care clinic in Toronto. The MLMC treats approximately 10,000 unique patients a year, approximately 80 % of whom are MSM (personal communication, R Halpenny). Recruitment occurred from September 2010 to June 2012. Eligibility criteria included being male, 16 years or older, having had sex with a man in the previous 12 months, and living in the Greater Toronto Area [23].

### Data collection

In the original study, primary care doctors informed MLMC patients of the study and then patients were approached by the study coordinator who provided study details. Participants were recruited based on their known, self-reported HIV status. If participants were eligible and willing to participate, written informed consent was obtained and an appointment was made to complete the questionnaire and obtain specimen collections. Survey questions included demographic and sexual behaviour characteristics and drug and alcohol use (Additional file 1). Study participants also provided blood, urine and a self-collected anal swab for the purpose of confirming self-reported HIV status, and testing for other STIs. Study participants completed a self-administered questionnaire using ACASI (Audio Computer Assisted Self-Interview; Questionnaire Development System (QDS) Version 2.5, Nova Research Company, Bethesda, Maryland, USA). Participants were compensated $50 for their time, travel, knowledge and biologic samples.

### Outcome and independent variables

The outcome variable was perceived risk of HIV infection. Participants were asked “what do you think the chances are that you will ever get HIV/AIDS?” This was asked on a four-point Likert scale and dichotomized into “high” (somewhat, very likely) versus “low” (impossible, not likely) perceived risk after looking at the variability of responses.

There is no one variable that defines actual sexual risk. Therefore, we set out to create a theoretical latent variable of actual risk using PCA that was represented by eight sexual risk manifest variables [22]: number of casual partners, number of regular partners, condom use during insertive anal sex with casual partners, condom use during receptive anal sex with casual partners, condom use during insertive anal sex with regular HIV-positive partner, condom use during receptive anal sex with regular HIV-positive partner, condom use during insertive anal sex with a regular HIV unknown status partners and condom use during receptive anal sex with a regular HIV-unknown status partner. All questions were asked in the context of the previous 6 months. The best PCA model was chosen based on five criteria: having an eigenvalue above 1.0, breaks in the scree plot, the cumulative proportion of variance accounted for above 70 %, the individual proportion of variance accounted for above 10 % and interpretability that included having a simple structure and that loadings made theoretical sense and had conceptual meaning [22]. A varimax orthogonal rotation was employed, and loading above 0.4 on only one component was considered meaningful. If a variable loaded above 0.4 on more than one component, it was considered to double load and was removed. When the final PCA was chosen, component scores were computed. Component scores are a linear composite of optimally weighted observed variables. The scores have a mean of zero and a variance of one [22]. Interpreting continuous component scores is difficult and open to interpretation. To give the scores more meaning, we used tertile cut points and labelled these high, medium and low scores. The Cronbach alpha at a cut-off of 0.7 was used to assess reliability [24].

A number of researchers have attempted to create a screening tool for physicians to use to decide whether HIV prevention services and pre-exposure prophylaxis (PrEP) use is appropriate [21, 25, 26]. These screening tools involve a short survey and based on answers, a score is given. Above a certain score, the patient has been engaging in high risk behaviour, and is a good candidate for intensive HIV prevention services. The seven question HIV Incidence Risk Index for MSM (HIRI-MSM) was chosen because it was specific to MSM, had high sensitivity and had been validated [21]. We wanted to determine if individuals who scored high on this index (a score of ten or more), and hence had a high risk for HIV acquisition, also had a high perceived risk. Therefore, a second multivariable logistic regression was run where the HIRI-MSM variable replaced the actual HIV risk PCA variables. All seven required variables to create the HIRI-MSM index score were in our dataset; however, one question was modified to fit the index and a score of six was changed to a score of three.

### Statistical analysis

Data were analyzed using SAS 9.3 (SAS Institute, Cary NC) and STATA 13 (StataCorp. 2013. *Stata Statistical Software: Release 13*. College Station, TX: StataCorp LP). Demographic and sexual behaviour characteristics of participants were described using median and inter-quartile ranges for continuous variables, and proportions for categorical variables. The relationship between actual risk (as defined by PCA) and perceived risk was modeled using multivariable logistic regression controlling for theorized confounders [27], including age, ethnicity, education, personal income, number of lifetime HIV tests, marital status, ever having had an STI, ever having used post-exposure prophylaxis (PEP), having used poppers in the previous six months, and having used methamphetamines or crystal meth in the previous 6 months. Model reduction techniques occurred before running the multivariable logistic model to ensure the final model was parsimonious and yielded non-biased p-values [27]. Odds ratios (OR) and 95 % confidence intervals (CI) are reported. Ethics approval for this analysis was obtained from the University of Toronto Research Ethics Board.

## Results

### Demographic profile

A total of 150 HIV-negative MSM were recruited, completed the questionnaire and provided biological samples. One hundred and forty five MSM answered the question about perceived risk and a total of 29 (20 %) had a high perceived risk of HIV infection. The median age was 44.5 years (IQR 37–50 years) and 83 % self-identified as white (Table 1). The majority of participants had undergraduate or graduate level education and almost half were single and never married. Seventy two percent of participants had ever been diagnosed with an STI and the majority had five or more HIV tests in their lifetime. Over one quarter of participants had taken poppers in the previous six months.

**Table 1:** Demographic and other characteristics of study participants by perceived risk and bivariate logistic regression of high versus low perceived risk

		Total^a^	Perceived risk		OR	95 % CI
Characteristics			Low (*n* = 116)	High (*n* = 29)		
		n(%)	n(%)	n(%)		
Age (years)	<30	9 (6.29)	5 (4.35)	4 (14.29)	1.00	
	30–39	36 (25.17)	28 (24.35)	8 (28.57)	0.41	0.093, 1.83
	40–49	57 (39.86)	47 (40.87)	10 (35.71)	0.38	0.093, 1.58
	50–59	32 (22.38)	28 (24.35)	4 (14.29)	0.21	0.041, 1.11
	60+	9 (6.29)	7 (6.09)	2 (7.14)	0.43	0.057, 3.22
Ethnicity	White	119 (84.40)	96 (85.71)	23 (79.31)	1.00	
	Other	22 (15.60)	16 (14.29)	6 (20.69)	1.66	0.62, 4.47
Education	High School or less	9 (6.21)	8 (6.90)	1 (3.45)	1.00	
	Some or completed undergraduate	103 (71.03)	81 (69.83)	22 (75.86)	1.42	0.29, 6.94
	Some or completed graduate	33 (22.76)	27 (23.28)	6 (20.69)	1.11	0.19, 6.44
Personal Income	$19,999 or less	35 (24.31)	25 (21.55)	10 (35.71)	1.00	
	$20,000–$59,999	60 (41.67)	49 (42.24)	11 (39.29)	0.60	0.23, 1.54
	$60,000–$99,999	31 (21.53)	26 (22.41)	5 (17.86)	0.47	0.14, 1.55
	$100,000 or more	18 (12.50)	16 (13.79)	2 (7.14)	0.31	0.060, 1.56
Lifetime number of HIV tests	Less than 5	27 (18.62)	25 (21.55)	2 (6.90)	1.00	
	5–9	44 (30.34)	33 (28.45)	11 (37.93)	2.89	0.73, 11.44
	10–19	41 (28.28)	32 (27.59)	9 (31.03)	2.36	0.58, 9.62
	20–29	23 (15.86)	17 (14.66)	6 (20.69)	3.06	0.67, 13.91
	30 or more	10 (6.90)	9 (7.76)	1 (3.45)	0.96	0.089, 10.48
Ever STI diagnosis	no	40 (27.59)	33 (28.45)	7 (24.14)	1.00	
	yes	105 (72.41)	83 (71.50)	22 (75.86)	1.33	0.52, 3.38
Ever taken post-exposure prophylaxis (PEP)	No	130 (90.28)	105 (91.30)	25 (86.21)	1.00	
	Yes	14 (9.72)	10 (8.70)	4 (13.79)	2.54	0.85, 7.66
Used amyl nitrite (poppers) in previous 6 months	No	104 (72.73)	91 (79.13)	13 (46.43)	1.00	
	Yes	39 (27.27)	24 (20.87)	15 (53.57)	3.88	1.66, 9.02
Used methamphetamines or crystal meth in previous 6 months	No	129 (90.21)	106 (92.17)	23 (82.14)	1.00	
	Yes	14 (9.79)	9 (7.83)	5 (17.86)	2.40	0.74, 7.78
Marital status	Married/common law to male or female	55 (38.19)	45 (38.79)	10 (35.71)	1.00	
	Divorced/separated/widowed from male or female	22 (15.28)	18 (15.52)	4 (14.29)	1.00	0.28, 3.60
	Single, never married	67 (46.53)	53 (45.69)	14 (50.00)	1.31	0.54, 3.17
Component 1: Condom use during insertive or receptive anal sex with casual male partner or number of casual partners	Low	49 (33.79)	42 (36.21)	7 (24.14)	2.29	0.82, 6.39
	Med	49 (33.79)	40 (34.48)	9 (31.03)	1.35	0.46, 3.97
	High	47 (32.41)	34 (29.31)	13 (44.83)	1.00	
Component 2: Condom use during insertive or receptive anal sex with an HIV-positive regular male partner	Low	48 (33.10)	40 (34.48)	8 (27.59)	1.60	0.62, 4.12
	Med	31 (21.38)	26 (22.41)	5 (17.24)	0.96	0.28, 3.62
	High	66 (45.52)	50 (43.10)	16 (55.17)	1.00	
Component 3: Condom use during insertive or receptive anal sex with an HIV-unknown status regular male partner	Low	49 (33.79)	38 (32.76)	11 (37.93)	2.07	0.71, 6.01
	Med	72 (49.66)	63 (54.31)	9 (31.03)	0.49	0.19, 1.30
	High	24 (16.55)	15 (12.93)	9 (31.03)	1.00	
HIRI-MSM score (continuous)	median (IQR)	10 (2–19)	9 (2–16)	18 (8–25)	1.07	1.02, 1.11
HIRI-MSM score	9 or less	68 (46.90)	60 (51.72)	8 (27.59)	1.00	
	10 or more	77 (53.10)	56 (48.28)	21 (72.41)	2.25	0.98, 5.18

^a^Number of subjects missing values or answering ‘don’t know’ or refuse to answer for age (2), ethnicity (4), personal income (1), PEP use (1), poppers (2), methamphetamines (2), marital status (1)

*OR* Odds Ratio, *95 % CI* 95 % Confidence Intervals, *STI* Sexually transmitted infection, *HIRI-MSM *HIV Risk Index for MSM

### Principal components analysis: actual risk

Three components accounted for 78.1 % of the total variance and were meaningful in the PCA analysis (Tables 2 and 3). The variable “number of regular partners” was dropped from the PCA due to double loading. For all three components, variables loaded above 0.4 for only one component, eigenvalues were above one, and proportional variance was above 10 %. The Cronbach coefficient alphas were all above the 0.7 recommended cut-off.

**Table 2:** Component loadings, of final PCA model

Variable	Component 1	Component 2	Component 3
Number of casual partners	88^a^	9	6
Condom use during insertive anal sex with casual partner	77^a^	6	25
Condom use during receptive anal sex with casual partner	74^a^	30	18
Condom use during insertive anal sex with regular HIV-positive partner	10	88^a^	18
Condom use during receptive anal sex with regular HIV-positive partner	22	89^a^	16
Condom use during insertive anal sex with regular HIV-unknown status partner	19	18	88^a^
Condom use during receptive anal sex with regular HIV-unknown status partner	18	17	87^a^

^a^variable significantly loaded onto component

**Table 3:** E igenvalues, variance accounted for, and reliability of final PCA model

	Eigenvalue	Proportion^a^	Cumulative^a^	Coefficient alpha^b^
Component 1	3.26	0.47	0.47	0.77
Component 2	1.19	0.17	0.64	0.82
Component 3	1.01	0.15	0.78	0.80

^a^proportion and cumulative variance accounted for in final PCA model

^b^standardized Cronbach coefficient alpha

Component I labeled “casual sex” comprised three variables: number of casual partners, condom use during insertive anal intercourse with a casual partner and condom use during receptive anal intercourse with a casual partner. A high score on component I (labeled low) corresponds to low condom use during insertive or receptive anal sex with a casual partner and a greater number of casual partners, whereas a low score on component I (labeled high) corresponds to high condom use during insertive or receptive anal sex with a casual partner and lower number of casual partners.

Component II labeled “HIV-positive sex” comprised two variables: condom use during insertive anal intercourse with a regular HIV-positive partner and condom use during receptive anal intercourse with a regular HIV-positive partner. A high score on component II (labeled low) corresponds to low condom use during insertive or receptive anal sex with an HIV-positive regular partner, whereas a low score on component II (labeled high) corresponds to high condom use during insertive or receptive anal sex with an HIV-positive regular partner.

Component III labeled “HIV unknown status sex” comprised two variables: condom use during insertive anal intercourse with a regular HIV unknown status partner and condom use during receptive anal intercourse with a regular HIV unknown status partner. A high score on component III (labeled low) corresponds to low condom use during insertive or receptive anal sex with an HIV unknown status regular partner, whereas a low score on component III (labeled high) corresponds to high condom use during insertive or receptive anal sex with an HIV unknown status regular partner.

### Bivariate regression

Bivariate logistic regression showed no significant associations between the outcome ‘perceived risk’ and any of the following theorized confounders: age, ethnicity, education, personal income, marital status, number of lifetime HIV tests, ever taken PEP or use of methamphetamines in the previous 6 months (Table 1). Furthermore, there was no significant association between perceived risk and any actual risk variables such as unprotected sex with a casual or regular partner, irrespective of HIV status of partner. Perceived risk was not significantly associated with the actual risk variables from the PCA. Perceived risk was significantly associated with a one point increase on the HIRI-MSM index (OR 1.07, 95 % CI 1.02–1.11) but not significant when scored high versus low risk (OR 2.25, 95 % CI 0.98–5.18). The only theoretical confounder associated with high perceived risk was the use of poppers in the previous 6 months (OR 3.88, 95 % CI 1.66–9.02).

### Multivariable regression

In the primary multivariable model, the odds of a high perceived risk was significantly higher for MSM with low condom use during insertive or receptive anal sex with a regular HIV-positive partner compared to MSM with a high condom use with HIV-positive regular partners (OR 18.33, 95 % CI 1.65–203.45) (Table 4). There was no significant association between perceived risk and level of condom use with casual partners or perceived risk and level of condom use with regular HIV-unknown status partners. Being 40–49 years old was statistically significantly associated with decreased perceived risk (OR 0.12, 95 % CI 0.016–0.86). There was an indication that being non-White was associated with a higher perceived risk (OR 4.18, 95 % CI 0.085–20.55), however this was not significant. The odds of having a high perceived risk was significantly increased in MSM who used poppers in the previous 6 months compared to MSM who did not use poppers (OR 5.64, 95 % CI 1.20–26.48).

**Table 4:** Multivariable logistic regression of variables associated with high versus low perceived risk

		Odds Ratio (95 % CI)
Component 1: Condom use during insertive or receptive anal sex with casual male partner or number of casual partners	Low	6.85 (0.20–239.61)
	Med	1.77 (0.11–28.50)
	High	1.00
Component 2: Condom use during insertive or receptive anal sex with an HIV-positive regular male partner	Low	18.33 (1.65–203.45)
	Med	4.16 (0.31–56.54)
	High	1.00
Component 3: Condom use during insertive or receptive anal sex with an HIV-unknown status regular male partner	Low	4.02 (0.52–30.83)
	Med	0.87 (0.087–8.71)
	High	1.00
Age	<30	1.00
	30–39	0.21 (0.029–1.51)
	40–49	0.12 (0.016–0.86)
	50–59	0.13 (0.013–1.28)
	60+	0.30 (0.015–5.95)
Ethnicity	White	1.00
	Other	4.18 (0.85–20.55)
Lifetime number of HIV tests	0–4	1.00
	5–9	3.87 (0.32–46.38)
	10–19	3.59 (0.32–39.74)
	20–29	8.11 (0.50–130.68)
	30 or more	0.38 (0.0080–18.028)
Education	High School or less	1.00
	Some or completed undergraduate	6.80 (0.33–140.19)
	Some or completed graduate	5.45 (0.16–189.71)
Personal income	$19,999 or less	1.00
	$20,000–$59,999	0.49 (0.12–2.06)
	$60,000–$99,999	0.33 (0.054–2.03)
	$100,000 or more	0.059 (0.0030–1.23)
Ever STI diagnosis	no	1.00
	yes	0.84 (0.21–3.39)
Ever taken post-exposure prophylaxis (PEP)	no	1.00
	Yes	0.50 (0.071–3.44)
Used amyl nitrite (poppers) in previous 6 months	No	1.00
	Yes	5.64 (1.20–26.48)
Used methamphetamines or crystal meth in previous 6 months	No	1.00
	Yes	0.66 (0.063–6.81)
Marital status	Married/common law to male or female	1.00
	Divorced/separated/widowed from male or female	0.53 (0.071–3.97)
	Single, never married	0.69 (0.16–2.98)
*N* = 134		

Over 53 % of participants scored 10 or above on the HIRI-MSM index indicating that physicians should speak to them about PrEP or other intensive HIV prevention services. In the secondary multivariable model, where the HIRI-MSM index replaced the PCA variables, we found that the HIRI-MSM variable, which is indicative of risk, was not significantly associated with a high perceived risk (OR 0.84, 95 % CI 0.21-3.42. The only variable that was significantly associated with perceived risk was the use of poppers in the previous 6 months (OR 6.23, 95 % CI 1.42-27.30) (data not shown).

## Discussion

We found that 20 % of HIV-negative MSM attending the MLMC in Toronto perceived themselves to be at high risk for HIV acquisition. While this is consistent with another Toronto clinic based MSM study where 17 % perceived their risk to be high [28], this proportion is significantly higher than the U.S. national average of 7.7 % [14]. Among the three actual risk components, only low versus high condom use during insertive or receptive anal sex with an HIV-positive regular partner was significantly associated with high perceived risk. It is hard to gauge whether this is in fact risky, and hence perceptions are correct, as we do not know the antiretroviral therapy (ART) status or viral load levels of the HIV-positive partners. Different levels of condom use during insertive or receptive anal sex with a regular HIV unknown status partner or a casual partner was not significantly associated with high perceived risk. With an estimated 18 % of HIV-positive MSM in Canada not knowing their status [1], this is perhaps the most concerning result. Condomless sex when status is not known or discussed means that individuals could be putting themselves at high risk for HIV acquisition without recognizing (perceiving) how risky the behaviour could be. It has been shown that when HIV-positive status is disclosed, there is significantly increased condom use [29].

The HIRI-MSM index and our PCA methods used very similar variables to measure HIV risk. However, the HIRI-MSM index incorporates popper and methamphetamine use along with high risk sexual behaviours. Since popper use was such a strong independent predictor of high perceived risk, we would have thought that the HIRI-MSM index would have had a strong, positive relationship with perceived risk. However, a higher HIRI-MSM index score indicating higher risk was not associated with perceived risk. The lack of association may indicate the need for more HIV education on risk behaviours in this population.

Popper use has been shown to be associated with increased unprotected sex [30, 31], increased sexual pleasure, and aiding in anal sex by relaxing the anal sphincter [7, 32, 33]. Given the increase in risk behaviour while taking this drug, it is not surprising that popper use was significantly associated with high perceived risk. It is however interesting to note that methamphetamine use was not associated with higher perceived risk, when it too has been shown to be associated with increased unprotected sex and increased number of sexual partners [34, 35]. This could be due to differences in drug effect, the situation in which the drug is taken, or prior intent of safer sex practices.

Many of the protective health behaviour models are based on the belief that educating people about the risk associated with behaviours will encourage them to reduce high risk behaviour in the future [36]. This reduction of risk over time could result in a change in the person’s risk perception. However, education may not be enough to reduce risk. Some studies have found that high HIV knowledge is not associated with reduced sexual risk behaviour [37]. This is why the protective health behaviour models also take into account the severity of the negative event, and the barriers to performing the preventive action when taking into account the motivation to change behaviour [10, 36]. With the advent of highly active antiretroviral therapy (HAART), HIV acquisition is seen as a less severe negative event. There has been an increase in sexual risk behaviour since HAART became available [38–40] and it has also been found that perceiving HIV as less of a threat, because of HAART, is associated with more risky behaviour [41–43]. Hence, perceived risk could be high, but not motivational enough to change behaviour. Also, because safe sex involves negotiation with a partner, there could be barriers to performing the preventive action that is out of the control of the individual. Altogether, this may disrupt the cyclical nature of actual and perceived risk.

As suggested earlier, there is no single question that can be asked to assess HIV risk, and there is no consensus from researchers and physicians on how best to categorize HIV risk. Because of this, there is no consistency between studies assessing actual and perceived risk. The HIRI-MSM index may have great value in physicians’ offices, where the patient can respond to each question, get their risk score, and open the discussion for possible prevention methods; however, not every study contains the information needed to create this score, and some studies want to look at sexual behaviour risk by itself. PCA allowed us to use all of the data from our study, while limiting in the number of variables in the multivariable model. We were able to assess actual sexual risk in the context of perceived risk while controlling for possible confounding.

Recruitment for this study occurred at a primary care and HIV clinic in downtown Toronto that caters to MSM. Therefore, results may not be generalized to all MSM in Toronto, but rather MSM in Toronto who are accessing health care. Persons accessing health care may already have a higher perceived risk, or health consciousness than those who do not access health care. We wanted to have an HIV knowledge composite score in the multivariable model because we theorized it would be an important confounder of the relationship between actual and perceived risk. We did not have appropriate HIV knowledge questions in our questionnaire to be able to make a composite score. Therefore, we may be missing an important confounder in our model. Due to the small sample size, our study is underpowered and may not detect otherwise statistically significant associations between high perceived risk and independent variables. The relationship between risk behaviour and perceived risk is thought to be cyclical. Since our study was cross-sectional, we are not able to determine causal or temporal associations. However, we did ask about sexual behaviour in the previous 6 months, and current perceived risk, which is how we determined our primary predictor and outcome.

## Conclusions

Overall, we found that one in five HIV-negative MSM in our study perceived their risk of HIV infection to be high. Participants who reported low versus high condom use with an HIV-positive regular partner were significantly more likely to report a high perceived risk, as were participants who reported using poppers in the previous 6 months. Other high risk behaviours such as low condom use with casual or HIV-unknown status regular partners, or methamphetamine use, were not associated with high perceived risk. Due to the high proportion of undiagnosed HIV-positive persons, and the nature of casual relationships (where HIV status may not be discussed), an education campaign that targets safer sex practices with these individuals is recommended.

## Additional file

Additional file 1: The epidemiology of co-infections in HIV affected communities from Toronto, Canada. Audio Computer-Assisted Self Interview (ACASI) Questionnaire. Men who have sex with men. (DOC 823 kb)

## Abbreviations

95 % CI: 95 % confidence intervals
ACASI: audio computer assisted self-interview
HIRI-MSM: HIV incidence risk index for MSM
HIV: human immunodeficiency virus
MLMC: maple leaf medical clinic
MSM: men who have sex with men
OR: odds ratio
PCA: principal components analysis
STIs: sexually transmitted infections
