# Supplementary material for: Actual sexual risk and perceived risk of HIV acquisition among HIV-negative men who have sex with men in Toronto, Canada
Source: BMC Public Health. 2016 Mar 11;16:254. doi: 10.1186/s12889-016-2859-6 (PMC4788863; doi:10.1186/s12889-016-2859-6)
Supplement: Additional file 1: — The epidemiology of co-infections in HIV affected communities from Toronto, Canada. Audio Computer-Assisted Self Interview (ACASI) Questionnaire. Men who have sex with men. (DOC 823 kb) [file 12889_2016_2859_MOESM1_ESM.doc]

**ADDITIONAL FILE 1:**

**THE EPIDEMIOLOGY OF CO-INFECTIONS IN HIV AFFECTED COMMUNITIES FROM TORONTO, CANADA**

**Audio Computer-Assisted Self Interview (ACASI) Questionnaire**

**Men who have sex with men**

**SECTION 1: INTERVIEW INFORMATION**

1. Subject ID: __________________ [SUBJECT]

(to be entered by the research coordinator, in format as following ‘MLMC0001’)

1. Date of interview (automatic variable) [TODAY]

Day: __________ Month: __________ Year: __________

[YRS1] (Automatic variable) = (TODAY - 1)/365.25

[CURYEAR] (Automatic variable) = truncate (1900 + YRS1)

1. Time interview started (Automatic variable) [STARTIME]

NOTE: ALL INFORMATION TO RESPONDENT IS IN ITALICS IN THIS QUESTIONNAIRE.

**Introduction**

*Thank you for your participation. This interview will collect information regarding your background and risk factors for sexually transmitted infections. It includes some questions on sexual behaviour and other behaviour that you may find sensitive and may wish not to answer. You are free to refuse to answer any question.*

*Please listen and follow the instructions on the screen. If you want to repeat the question, click the "****Repeat the question****" button.*

*You can move the mouse to choose your answer OR type letters and numbers using the keyboard.*

*When you enter an answer in a text box, use the keyboard (lower and upper case letters are both fine).*

*If you wish to use the letter or number buttons on the screen, click the “Alt” button to switch the letter button screen to the number button screen and vice versa.* [Info1a]

*If you realize that you entered a wrong answer on a question, you may click the "****Previous question****" button and go back to the question that you wish to change.*

*If you do not remember or do not know how to answer a question, you may click the "****Don’t know****" button.*

*If you have any questions during the interview,* ***please contact Ms. Molly Gamble for assistance****.* [Info1b]

**SECTION 2: SOCIO-DEMOGRAPHIC CHARACTERISTICS**

*First, we need some information about you. This information is for research purposes only and will be kept strictly confidential. No personal identifiers are kept in the computer file.*  [Info2]

1. What is your date of birth? *(If you don’t wish to enter your full date of birth, please enter the year only)*  [DOB]

Day: __________ Month: __________ Year: __________

*[Range: 1929/01/01-1994/12/31 (age 16 to 80 years old)]*

1. Don’t know
2. Refuse to answer
3. What was your sex at birth? [SEXBORN]
4. Male
5. Female
6. Don’t know
7. Refuse to answer
8. Where do you currently live? ***(click only one answer)*** [RESIDE]
9. Ontario **🡪 go to question 6.1**
10. British Columbia
11. Alberta
12. Saskatchewan
13. Manitoba
14. Quebec
15. New Brunswick
16. Nova Scotia
17. Prince Edward Island
18. Newfoundland and Labrador
19. Northwest Territories
20. Yukon
21. Nunavut
22. Outside Canada **🡪 go to question 6.3**
23. Don’t know
    1. In which city, town or district do you currently live? ***(click only one answer)***

[RESIDE1]

1. Toronto
2. Mississauga
3. Brampton
4. Oakville
5. Vaughan
6. Markham
7. Pickering
8. Ajax
9. Whitby
10. Oshawa
11. Hamilton
12. Kitchener
13. London
14. Windsor
15. Ottawa
16. Richmond Hill
17. Other **🡪 go to question 6.2**
18. Don’t know
19. Refuse to answer

**🡪 go to question 7**

- 1. Please specify the city, town or district that you currently live in *(Use the keyboard to type or click letters on the screen)* [RESIDE2]

_________________

- Don’t know
- Refuse to answer

**🡪 go to question 7**

- 1. Please enter the country *(Use the keyboard to type or click letters on the screen)* [RESIDE3]

_________________

- Don’t know
- Refuse to answer

**🡪 go to question 8**

1. Ignore the letters below, type the first three characters of your postal code in Canada *(in 'letter-number-letter' format, for example: M4C)* [PCODE]

__ __ __

- Don’t know **🡪 go to question 8**
- Refuse to answer **🡪 go to question 8**

[PCODEC1] (Automatic variable)

[PCODEC2] (Automatic variable)

[PCODEC3] (Automatic variable)

**[Check: format of postal code, if not the correct format**

**🡪 loop back to question 7]**

**Message:**

The first character should be a letter, please correct it.

The second character should be a number, please correct it.

The third character should be a letter, please correct it.

1. What is the highest level of education that you have completed? ***(click only one answer)***

[EDU]

1. No education
2. Some elementary/ primary school
3. Completed elementary/ primary school
4. Some secondary/high school
5. Completed secondary/high school
6. Some college or university
7. Completed college or university
8. Some graduate education (e.g. Masters, PhD, MD programs)
9. Completed graduate education (e.g. Masters, PhD, MD degrees)
10. Don’t know
11. Refuse to answer

**Country of birth, if not Canada, immigration history**

*The next few questions ask about your country of birth and immigration history.* [Info3]

1. In what country/region were you born? [COB]
2. Canada **🡪 go to question 10**
3. Other
4. Don’t know **🡪 go to question 10**
5. Refuse to answer **🡪 go to question 10**
   1. Please specify the country you were born in *(Use the keyboard to type or click letters on the screen)* [COB1]

______________________

- Don’t know
- Refuse to answer
  1. Time arrival of Canada
     1. In what year did you first come to Canada to live? [COB2A]

________(year)

1. Don’t know
2. Refuse to answer

**[Check: should be later than year of birth, as well as, before date of interview 🡪 loop back to question 9.2a]**

**Message:**

The year of your arrival to Canada should be later than the year of your birth, as well as, before today. Please correct it.

If Don’t know or Refuse to answer,

- - 1. How old were you when you first arrived in Canada? [COB2B]

________ (age in years) (range 0 -80)

1. Don’t know
2. Refuse to answer

**[Check: should be younger than or equal to current age**

**🡪 loop back to question 9.2b]**

**Message:**

Your age when you arrived in Canada should be younger than your current age. Please correct it.

If year of arrival of Canada is known, then

[ARVYEAR] (Automatic variable) = COB2A

If age of arrival of Canada is known, then

[ARVYEAR] (Automatic variable) = CURYEAR – (AGE – COB2B)

If age of arrival of Canada is unknown or Refuse to answer, then

[ARVYEAR] (Automatic variable) = -1 (missing)

- 1. Where was the last place you were living immediately before coming to Canada?

[LASTPL1]

1. Same as your country of birth
2. Other country **🡪 go to question 9.3a**
3. Don’t know
4. Refuse to answer
   - 1. Please indicate the country *(Use the keyboard to type or click letters on the screen)* [LASTPL2]

______________

- Don’t know
- Refuse to answer
  1. What was your immigration status when you first arrived in Canada? ***(click only one answer)*** [IMMARV]

1. Landed/permanent resident
   1. How did you acquire permanent residency? ***(click only one answer)***

[IMMARVA]

1. Sponsored by family
2. Economic immigrant (independent/skilled worker/live-in caregiver)
3. Refugee/person in need of protection
4. Don’t know
5. Refuse to answer

**🡪 go to question 9.5**

1. Refugee claimant/person in need of protection (before decision had been made by immigration authorities)
2. Temporary worker
3. Visitor
4. Student
5. Non-status
6. Don’t know
7. Refuse to answer
   1. What is your current immigration status in Canada? ***(click only one answer)***

[IMMCUR]

1. Canadian citizen
2. Landed/permanent resident
3. Refugee (immigration authorities have approved your status as a refugee)
4. Refugee claimant/person in need of protection (immigration authorities have not yet made a decision about your refugee status, or they have Refuse to answer your application for refugee status and you have applied again)
5. Temporary worker
6. Visitor
7. Student
8. Non-status
9. Don’t know
10. Refuse to answer

**Religion, language and culture**

*The next few questions ask about your religion, language and ethnicity.* [Info4]

1. What is your faith or religion? ***(click only one answer)*** [RELIG]
2. None/agnostic/atheist **🡪 go to question 11**
3. Christian
4. Jewish **🡪 go to question 11**
5. Muslim
6. Hindu **🡪 go to question 11**
7. African traditional
8. Buddhism **🡪 go to question 11**
9. Other
10. Don’t know **🡪 go to question 11**
11. Refuse to answer **🡪 go to question 11**

If Christian, Muslim, African traditional, or other

- 1. Please specify the type of your religion *(For example, Catholic in Christian, Sunni in Muslim, etc) (Use the keyboard to type or click letters on the screen)* [RELIG1]

______________________________

- Don’t know
- Refuse to answer

1. What is your ethnic/cultural background? ***(click all that apply)*** [ETHNIC]
   - 1. White (Caucasian)
     2. Black African born in Canada
     3. Black African born outside Canada
     4. Black Caribbean born in Canada
     5. Black Caribbean born outside Canada
     6. East/Southeast Asian (e.g. Chinese, Japanese, Korean, Vietnamese, Cambodian, Indonesian, Laotian, Filipino, etc)
     7. South Asian (e.g. East Indian, Pakistani, Sri Lankan, Punjabi, Bangladeshi, etc)
     8. Arab/West Asian (e.g. Armenian, Egyptian, Iranian, Lebanese, Moroccan, Israeli, etc)
     9. Latin American (e.g. Mexican, Central/South American, etc)
     10. Aboriginal people (e.g. Native Indian/First Nation, Inuit or Métis)
     11. Other (including mixed ethnicity) **🡪 go to question 11.1**
2. Don’t know
3. Refuse to answer

If other,

- 1. Please specify your ethnic/cultural background *(Use the keyboard to type or click letters on the screen)*  [ETHIC1] ________________________
- Don’t know
- Refuse to answer

1. Questions about language:
   1. What is the first language you first learned and still understand? ***(click all that apply)*** [FSTLANG]
2. English
3. French
4. Other **🡪 go to question 12.1a**
5. Don’t know
6. Refuse to answer

If other,

- - 1. Please specify your first language other than English and French *(Use the keyboard to type or click letters on the screen)* [FSTLANG1]

________________________

- Don’t know
- Refuse to answer
  1. Which languages do you speak fluently? *(By fluently, we mean languages that you would have no difficulty speaking)* ***(click all that apply)*** [LANG1]

1. English
2. French
3. Spanish
4. Other
5. Don’t know
6. Refuse to answer

If other,

- - 1. Please specify the languages other than English, French and Spanish that you can **speak** fluently *(List up to 3,* ***separate them by a comma ",";***  *for example, German, Mandarin, Portuguese) (Use the keyboard to type or click letters on the screen)* [LANG2]

________________________

- Don’t know
- Refuse to Answer
  1. Which languages do you read fluently? *(By fluently, we mean languages that you would have no difficulty reading)*  ***(click all that apply)*** [LANG3]

1. English
2. French
3. Spanish
4. Other
5. Don’t know
   1. Refuse to answer

If other,

- - 1. Please specify the languages other than English, French and Spanish that you can **read** fluently *(List up to 3,* ***separated by a comma ",";*** *for* *example, German, Mandarin, Portuguese) (Use the keyboard to type or click letters on the screen)*  [LANG4]

________________________

- Don’t know
- Refuse to Answer

**Family and living situation**

*The next few questions ask about your family and living situation* [Info5]

1. What is your current marital status? ***(click only one answer)*** [MRTL]
2. Married and living with wife (female partner)
3. Married and living with male partner
4. Common-law (female partner)
5. Common-law (male partner)
6. Divorced from female partner
7. Divorced from male partner
8. Separated from female partner
9. Separated from male partner
10. Widowed (female partner died)
11. Widowed (male partner died)
12. Single and never married
13. Don’t know
14. Refuse to answer
15. Have you fathered any children? *(By this, we mean that you are their biological father.)* [CHILD]
16. No **🡪 go to question 15**
17. Yes
18. Don’t know
19. Refuse to answer **🡪 go to question 15**

If yes,

- 1. How many children have you fathered? [CHILD1]

_________ (Range 1 - 99)

1. Don’t know
2. Refuse to answer
   1. How many of these children live with you currently? [CHILD2]
3. None
4. Some of them
5. All of them
6. Don’t know
7. Refuse to answer

1. What kind of housing do you live in now? ***(click only one answer)*** [HOUSE]
2. Single detached house
3. Semi-detached or double house
4. Townhouse or row house (attached to other houses)
5. Duplex (one above the other)
6. Low-rise apartment (fewer than 5 stories)
7. High-rise apartment (5 or more stories or condo)
8. Student residence or dorm
9. Hotel or rooming-house
10. Shelter/hostel (e.g. YMCA, Salvation Army)
11. Squat (an abandoned or unoccupied space or building)
12. No fixed address
13. Other **🡪 go to question 15.1**
14. Don’t know
15. Refuse to answer
    1. Please specify what kind of housing you live in *(Use the keyboard to type or click letters on the screen)* [HOUSE1]

___________________________

- Don’t know
- Refuse to answer

1. Including yourself, how many people live in your household currently? [HOUSE2]

_________ (range 1 – 99)

1. Don’t know
2. Refuse to answer

**[If only 1 🡪 go to question 18]**

1. How are they related to you? ***(click all that apply)***  [MEMB]
2. Spouse, opposite-sex as you
3. Spouse, same-sex as you
4. Opposite-sex common-law partner
5. Same-sex common-law partner
6. Parent If checked, **🡪 go to question 17.1**
7. Child If checked, **🡪 go to question 17.2**
8. Brother/sister If checked, **🡪 go to question 17.3**
9. Grandparent If checked, **🡪 go to question 17.4**
10. Grandchild If checked, **🡪 go to question 17.5**
11. In-law If checked, **🡪 go to question 17.6**
12. Other relative (e.g. uncle, aunt, cousins) If checked, **🡪 go to question 17.7**
13. Lodger or boarder If checked, **🡪 go to question 17.8**
14. Roommate/friend If checked, **🡪 go to question 17.9**
15. None of the above
16. Don’t know
17. Refuse to answer
    1. How many parents do you live with? [MEMBE1]

____________

1. Don’t know
2. Refuse to answer
   1. How many children do you live with? [MEMBF1]

____________

1. Don’t know
2. Refuse to answer
   1. How many brothers/sisters do you live with? [MEMBG1]

____________

1. Don’t know
2. Refuse to answer
   1. How many grandparents do you live with? [MEMBH1]

____________

1. Don’t know
2. Refuse to answer
   1. How many grandchildren do you live with? [MEMBI1]

____________

1. Don’t know
2. Refuse to answer
   1. How many in-laws do you live with? [MEMBJ1]

____________

1. Don’t know
2. Refuse to answer
   1. How many other relatives (e.g. uncles, aunts, cousins) do you live with? [MEMBK1]

____________

1. Don’t know
2. Refuse to answer
   1. How many lodgers or boarders live in your household? [MEMBL1]

____________

1. Don’t know
2. Refuse to answer
   1. How many roommates/friends live in your household? [MEMBM1]

____________

1. Don’t know
2. Refuse to answer

**Employment and income**

*The next few questions ask about your employment and income.* [Info6]

1. **During the past 12 months,** did you work at a job or business at any time in Canada? *(This includes part-time jobs, seasonal work, contract work, self-employment, baby-sitting and any other paid work regardless of the number of hours worked and whether you were paid a salary or in cash.)* [WORK]
2. No **🡪 go to question 20**
3. Yes
4. Don’t know **🡪 go to question 20**
5. Refuse to answer **🡪 go to question 20**
6. What is your work or occupation in Canada? *(Please be specific, for example, child-care worker,* *legal secretary,**wood furniture assembler, plumber,* *restaurant manager, secondary school teacher, sales and service occupations, etc.)* *(Use the keyboard to type or click letters on the screen)*  [OCCUP]

_____________

- Don’t know
- Refuse to answer

1. Which of these descriptions apply to your pattern of work last week, that is, in the past seven days ending last Sunday? ***(click only one answer)*** [WORKLWK]
2. Going to school, college, or university full-time (including on vacation)
3. Working for salary or self-employed
4. On vacation, sick leave, on strike, or absent for other reasons with pay
5. Working ‘under the table’ (i.e. cash basis)
6. Working without pay (voluntary work)
7. Unemployed
8. Unable to work because of long-term sickness or disability or on Ontario Disability Support Program (ODSP)
9. Looking after the home or family (i.e. caring for own children, elder relatives)
10. Retired
11. Doing something else **🡪 go to question 20.1**
12. Don’t know
13. Refuse to answer
    1. Please specify what were you doing last week *(Use the keyboard to type or click letters on the screen)*  [WORKLWK1]

___________________________

- Don’t know
- Refuse to answer

1. **During the past 12 months,** what is your estimated total personal income, before taxes and deductions, from all sources? *(Money received from Ontario Works (OW) and Ontario Disability Support Program (ODSP) is also considered as income)*  [INCOMEP]
2. No personal income
3. $1 - $9,999
4. $10,000 - $19,999
5. $20,000 - $29,999
6. $30,000 - $39,999
7. $40,000 - $49,999
8. $50,000 - $59,999
9. $60,000 - $69,999
10. $70,000 - $79,999
11. $80,000 - $89,999
12. $90,000 - $99,999
13. $100,000 or more
14. Don’t know
15. Refuse to answer
16. **During the past 12 months,** what is the estimated total income, before taxes and deductions, of all household family members including yourself from all sources? *(Money received from Ontario Works (OW) and Ontario Disability Support Program (ODSP) is also considered as income)*  [INCOMEF]
17. No family income
18. $1 - $9,999
19. $10,000 - $19,999
20. $20,000 - $29,999
21. $30,000 - $39,999
22. $40,000 - $49,999
23. $50,000 - $59,999
24. $60,000 - $69,999
25. $70,000 - $79,999
26. $80,000 - $89,999
27. $90,000 - $99,999
28. $100,000 or more
29. Don’t know
30. Refuse to answer

**Social activities**

*The next questions are about where and how you socialize.*  [Info7]

1. Which of the following types of activities or groups have you participated in **during the past 6 months**? ***(click all that apply)*** [EVENT]
2. Meetings of organizations (political, social, etc.) for gays and/or lesbians
3. Community events (e.g., parade, fair, fund-raiser, etc.) for gays and/or lesbians
4. Volunteer work for an organization for gays and/or lesbians
5. Called a help line for men who have sex with men
6. None of the above
7. Don’t know
8. Refuse to answer
9. Social places:
   1. How often do you go to a **gay** bar? [SCPLACE1]
10. Never
11. Once or twice a year
12. Less than once a month
13. 1 to 3 times a month
14. 1 to 2 times a week
15. 3 or more times a week
16. Don’t know
17. Refuse to answer
    1. How often do you go to a “**straight**” bar? [SCPLACE2]
18. Never
19. Once or twice a year
20. Less than once a month
21. 1 to 3 times a month
22. 1 to 2 times a week
23. 3 or more times a week
24. Don’t know
25. Refuse to answer
    1. How often do you go to a **bathhouse**? [SCPLACE3]
26. Never
27. Once or twice a year
28. Less than once a month
29. 1 to 3 times a month
30. 1 to 2 times a week
31. 3 or more times a week
32. Don’t know
33. Refuse to answer
    1. How often do you go to **gay dances, events or parties**? [SCPLACE4]
34. Never
35. Once or twice a year
36. Less than once a month
37. 1 to 3 times a month
38. 1 to 2 times a week
39. 3 or more times a week
40. Don’t know
41. Refuse to answer
42. Race discrimination:
    1. Have you ever felt uncomfortable in a gay bar or club because of your race or ethnicity? How often? [RCDISCR1]
43. Never
44. Once or twice
45. A few times
46. Many times
47. Don’t know
48. Refuse to answer
    1. Have you ever had trouble finding a boyfriend because of your race or ethnicity? How often? [RCDISCR2]
49. Never
50. Once or twice
51. A few times
52. Many times
53. Don’t know
54. Refuse to answer
    1. Have you ever felt that male sex partners paid more attention to you because of your race or ethnicity? How often? [RCDISCR3]
55. Never
56. Once or twice
57. A few times
58. Many times
59. Don’t know
60. Refuse to answer
    1. Have you ever felt that someone turned you down for sex because of your race or ethnicity? How often? [RCDISCR4]
61. Never
62. Once or twice
63. A few times
64. Many times
65. Don’t know
66. Refuse to answer
    1. Have you ever felt that your choice of a sex partner was less because of your race or ethnicity? How often? [RCDISCR5]
67. Never
68. Once or twice
69. A few times
70. Many times
71. Don’t know
72. Refuse to answer

**SECTION 3: SEXUAL HISTORY AND BEHAVIOUR**

*The following questions are about your sexual behaviour. In this study,* ***sex*** *means any activity, with or without orgasm, which involves the sexual organs, oral sex, and/or intercourse.* [Info8]

1. At the present time, how would you define yourself in terms of your sexual orientation? ***(click one best answer)*** [SEXDEF]
2. Gay or Homosexual
3. Bisexual
4. Straight or heterosexual
5. Two-spirited
6. Transsexual
7. Other **🡪 go to question 26.1**
8. Don’t know
9. Refuse to answer
   1. Please specify what would you define yourself in terms of your sexual orientation *(Use the keyboard to type or click letters on the screen)*  [SEXDEF1]

___________________________

- Don’t know
- Refuse to answer

1. What is your gender identity? ***(click one best answer)*** [SEXIDEN]
2. Man
3. Transwoman (male to female)
4. Transman (female to male)
5. Two-spirited
6. Woman
7. Intersex (i.e. both sex organs)
8. Other **🡪 go to question 27.1**
9. Don’t know
10. Refuse to answer
    1. Please specify what would you define yourself in terms of your gender identity *(Use the keyboard to type or click letters on the screen)*  [SEXIDEN1]

___________________________

- Don’t know
- Refuse to answer

***Sex with women***

*The following questions ask about your sex with women.* [Info9]

1. Have you **ever** had sex with a woman? [SEXWOMEN]
2. No **🡪 go to question**  **36**
3. Yes
4. Don’t know **🡪 go to question**  **36**
5. Refuse to answer **🡪 go to question**  **36**
6. How old were you the **first time** you had a sex with a woman? *(if you don't remember or don't know, click the “Do not know” button)* [FSTAGEW]

________ (age in years) (range 0 -70)

- 1. Don’t know

1. Refuse to answer

**[Check: should be younger than or equal to current age**

**🡪 loop back to question 29]**

**Message:**

You have indicated that your age when you first had sexual intercourse with a woman is older than you are now. Please correct it.

1. How old were you when you began having sex **on a regular basis** with a woman? *(At least once a month, but not necessarily with the same woman)* *(If never, please enter “0”)*

[AGEREGW]

________ (age in years) (range 0 -70)

- 1. Don’t know

1. Refuse to answer

**[Check: should be younger than or equal to current age**

**🡪 loop back to question 30]**

**Message:**

You have indicated that your age when you had sex on a regular basis with a woman is older than you are now. Please correct it.

1. **In your lifetime**, with how many **different women** have you had sex? (*vaginal, oral or anal sex, including women you may have had sex with only once*) *If you don’t remember or don’t know exactly, please click the “Don’t know” button.*  [NWPTNL]

___________ (number of women, range 1- 999)

1. Don’t know (Don’t remember) **🡪 go to question 31.1**
2. Refuse to answer **🡪 go to question 31.1**

If Don’t know or Refuse to answer,

- 1. If you don’t remember or don’t know exactly, check one of the following that best fits your situation: [NWPTNL1]

1. 1
2. 2 to 4
3. 5 to 9
4. 10 to 19
5. 20 to 49
6. 50 or more
7. Don’t know (Don’t remember)
8. Refuse to answer
9. **During the past 6 months**, have you had sex with a woman? [SEXW6M]
10. No **🡪 go to question 36**
11. Yes
12. Don’t know **🡪 go to question** **36**
13. Refuse to answer **🡪 go to question 36**
14. **During the past 6 months**, with how many **different women** have you had sex? (*vaginal, oral or anal sex, including women you may have had sex with only once) If you don’t remember or don’t know exactly, please click the “Don’t know” button.*

[NWPTN6M]

___________ (number of women, range 1- 999)

1. Don’t know (Don’t remember) **🡪 go to question 33.1**
2. Refuse to answer **🡪 go to question 33.1**

If Don’t know or Refuse to answer,

- 1. If you don’t remember or don’t know exactly, check one of the following that best fits your situation: [NWPTN6M1]

1. 1
2. 2 to 4
3. 5 to 9
4. 10 to 19
5. 20 to 49
6. 50 or more
7. Don’t know (Don’t remember)
8. Refuse to answer
9. Did you use a condom the last time you had sex with a woman? [LASTCNDW]
10. No
11. Yes
12. Don’t know
13. Refuse to answer
14. You indicated that you had sex with a woman **during the past 6 months;** how often did you use a condom? [CND6MW]
15. Never (0%)
16. Rarely (less than 25% of the time)
17. Sometimes (25-49% of the time)
18. Most of the time (50-74% of the time)
19. Almost every time (75-99% of the time)
20. All the time (100% of the time)
21. Don’t know
22. Refuse to answer

***Sex with men***

*The following questions ask about your sex with men.* [Info10]

1. Have you **ever** had sex with a man (oral or anal)? [SEXMEN]
2. No **🡪 go to question**  **62**
3. Yes
4. Don’t know **🡪 go to question**  **62**
5. Refuse to answer **🡪 go to question**  **62**
6. How old were you the **first time** you had a sex with a man? *(if you don't remember or don't know, click the “Do not know” button)* [FSTAGEM]

________ (age in years) (range 0 -70)

- 1. Don’t know

1. Refuse to answer

**[Check: should be younger than or equal to current age**

**🡪 loop back to question 37]**

**Message:**

You have indicated that your age when you first had sex with a man is older than you are now. Please correct it.

1. How old were you when you began to have sex **on a regular basis** with a man? *(At least once a month, but not necessarily with the same man)* *(If never, please enter “0”)*

[AGEREGM]

________ (age in years) (range 0 -70)

- 1. Don’t know

1. Refuse to answer

**[Check: should be younger than or equal to current age**

**🡪 loop back to question 38]**

**Message:**

You have indicated that your age when you had sex on a regular basis with a man is older than you are now. Please correct it.

1. **In your lifetime**, with how many **different men** have you had sex? *(oral or anal, including men you may have had sex with only once) If you don’t remember or don’t know exactly, please click the “Don’t know” button.* [NMPTNL]

___________ (number of men, range 1- 999)

1. Don’t know (Don’t remember) **🡪 go to question 39.1**
2. Refuse to answer **🡪 go to question 39.1**

If Don’t know or Refuse to answer,

- 1. If you don’t remember or don’t know exactly, check one of the following that best fits your situation: [NMPTNL1]

1. 1
2. 2 to 4
3. 5 to 9
4. 10 to 19
5. 20 to 49
6. 50 or more
7. Don’t know (Don’t remember)
8. Refuse to answer
9. **During the past 6 months**, have you had sex with a man (oral or anal)? [SEXM6M]
10. No **🡪 go to question 62**
11. Yes
12. Don’t know **🡪 go to question** **62**
13. Refuse to answer **🡪 go to question 62**
14. Places looking for sex with men:
15. During the past 6 months, have you looked for sex with men in a **gay bar**? How often? [FNDPLCA]
16. Never
17. Less than once a month
18. Once a month
19. 2 to 3 times a month
20. 1 to 2 times a week
21. 3 or more times a week
22. Don’t know
23. Refuse to answer
24. During the past 6 months, have you looked for sex with men in a **straight bar**? How often? [FNDPLCB]
25. Never
26. Less than once a month
27. Once a month
28. 2 to 3 times a month
29. 1 to 2 times a week
30. 3 or more times a week
31. Don’t know
32. Refuse to answer
33. During the past 6 months, have you looked for sex with men in a **rave/circuit party**? How often? [FNDPLCC]
34. Never
35. Less than once a month
36. Once a month
37. 2 to 3 times a month
38. 1 to 2 times a week
39. 3 or more times a week
40. Don’t know
41. Refuse to answer
42. During the past 6 months, have you looked for sex with men in a **bathhouse/sauna**? How often? [FNDPLCD]
43. Never
44. Less than once a month
45. Once a month
46. 2 to 3 times a month
47. 1 to 2 times a week
48. 3 or more times a week
49. Don’t know
50. Refuse to answer
51. During the past 6 months, have you looked for sex with men in an **after-hours club/party**? How often? [FNDPLCE]
52. Never
53. Less than once a month
54. Once a month
55. 2 to 3 times a month
56. 1 to 2 times a week
57. 3 or more times a week
58. Don’t know
59. Refuse to answer
60. During the past 6 months, have you looked for sex with men in a **private sex party**? How often? [FNDPLCF]
61. Never
62. Less than once a month
63. Once a month
64. 2 to 3 times a month
65. 1 to 2 times a week
66. 3 or more times a week
67. Don’t know
68. Refuse to answer
69. During the past 6 months, have you looked for sex with men in a **park or cruising area**? How often? [FNDPLCG]
70. Never
71. Less than once a month
72. Once a month
73. 2 to 3 times a month
74. 1 to 2 times a week
75. 3 or more times a week
76. Don’t know
77. Refuse to answer
78. During the past 6 months, have you looked for sex with men in a **public restroom**? How often? [FNDPLCH]
79. Never
80. Less than once a month
81. Once a month
82. 2 to 3 times a month
83. 1 to 2 times a week
84. 3 or more times a week
85. Don’t know
86. Refuse to answer
87. During the past 6 months, have you looked for sex with men in a **coffee shop**? How often? [FNDPLCI]
88. Never
89. Less than once a month
90. Once a month
91. 2 to 3 times a month
92. 1 to 2 times a week
93. 3 or more times a week
94. Don’t know
95. Refuse to answer
96. During the past 6 months, have you looked for sex with men on **internet (chat rooms)**? How often? [FNDPLCJ]
97. Never
98. Less than once a month
99. Once a month
100. 2 to 3 times a month
101. 1 to 2 times a week
102. 3 or more times a week
103. Don’t know
104. Refuse to answer
105. During the past 6 months, have you looked for sex with men on a **bareback website**? How often? [FNDPLCK]
106. Never
107. Less than once a month
108. Once a month
109. 2 to 3 times a month
110. 1 to 2 times a week
111. 3 or more times a week
112. Don’t know
113. Refuse to answer
114. During the past 6 months, have you looked for sex with men on a **telephone chat line**? How often? [FNDPLCL]
115. Never
116. Less than once a month
117. Once a month
118. 2 to 3 times a month
119. 1 to 2 times a week
120. 3 or more times a week
121. Don’t know
122. Refuse to answer
123. During the past 6 months, have you looked for sex with men on **personal ads**? How often? [FNDPLCM]
124. Never
125. Less than once a month
126. Once a month
127. 2 to 3 times a month
128. 1 to 2 times a week
129. 3 or more times a week
130. Don’t know
131. Refuse to answer
132. During the past 6 months, have you looked for sex with men in **community organizations**? How often? [FNDPLCN]
133. Never
134. Less than once a month
135. Once a month
136. 2 to 3 times a month
137. 1 to 2 times a week
138. 3 or more times a week
139. Don’t know
140. Refuse to answer
141. During the past 6 months, have you looked for sex with men in **gay associations**? How often? [FNDPLCO]
142. Never
143. Less than once a month
144. Once a month
145. 2 to 3 times a month
146. 1 to 2 times a week
147. 3 or more times a week
148. Don’t know
149. Refuse to answer
150. During the past 6 months, have you looked for sex with men in **recreational groups**? How often? [FNDPLCP]
151. Never
152. Less than once a month
153. Once a month
154. 2 to 3 times a month
155. 1 to 2 times a week
156. 3 or more times a week
157. Don’t know
158. Refuse to answer
159. During the past 6 months, have you looked for sex with men in a **gym/health club**? How often? [FNDPLCQ]
160. Never
161. Less than once a month
162. Once a month
163. 2 to 3 times a month
164. 1 to 2 times a week
165. 3 or more times a week
166. Don’t know
167. Refuse to answer
168. During the past 6 months, have you looked for sex with men in **community events for gays and lesbians**? How often? [FNDPLCR]
169. Never
170. Less than once a month
171. Once a month
172. 2 to 3 times a month
173. 1 to 2 times a week
174. 3 or more times a week
175. Don’t know
176. Refuse to answer
177. During the past 6 months, have you looked for sex with men in a **shopping mall**? How often? [FNDPLCS]
178. Never
179. Less than once a month
180. Once a month
181. 2 to 3 times a month
182. 1 to 2 times a week
183. 3 or more times a week
184. Don’t know
185. Refuse to answer
186. During the past 6 months, have you looked for sex with men in a **bookshop/theatre/video club**? How often? [FNDPLCT]
187. Never
188. Less than once a month
189. Once a month
190. 2 to 3 times a month
191. 1 to 2 times a week
192. 3 or more times a week
193. Don’t know
194. Refuse to answer
195. During the past 6 months, have you ever looked for sex with men in **other places not mentioned previously**? [FNDPLCU]
196. No  **🡪 go to question 42**
197. Yes
198. Don’t know
199. Refuse to answer

If yes,

- - 1. Please specify the other places where you have looked for sex with men during the past 6 months? *(Use the keyboard to type or click letters on the screen)*  [FINDPLC1]

___________________________

- Don’t know
- Refuse to answer
  - 1. How often have you looked for sex with men in this place during the past 6 months? [FINDPLC2]

1. Never
2. Less than once a month
3. Once a month
4. 2 to 3 times a month
5. 1 to 2 times a week
6. 3 or more times a week
7. Don’t know
8. Refuse to answer
9. Places having sex with men:
10. During the past 6 months, of all the times you had sex with another man, what proportion were in **your home**? [SEXPLCA]
11. Never
12. Sometimes (<30% of the time)
13. Often (30-69% of the time)
14. Usually (70-95% of the time)
15. Always (>95% of the time)
16. Don’t know
17. Refuse to answer
18. During the past 6 months, of all the times you had sex with another man, what proportion were in **his home**? [SEXPLCB]
19. Never
20. Sometimes (<30% of the time)
21. Often (30-69% of the time)
22. Usually (70-95% of the time)
23. Always (>95% of the time)
24. Don’t know
25. Refuse to answer

1. During the past 6 months, of all the times you had sex with another man, what proportion were in **private sex parties**? [SEXPLCC]
2. Never
3. Sometimes (<30% of the time)
4. Often (30-69% of the time)
5. Usually (70-95% of the time)
6. Always (>95% of the time)
7. Don’t know
8. Refuse to answer
9. During the past 6 months, of all the times you had sex with another man, what proportion were in **hotels or resorts**? [SEXPLCD]
10. Never
11. Sometimes (<30% of the time)
12. Often (30-69% of the time)
13. Usually (70-95% of the time)
14. Always (>95% of the time)
15. Don’t know
16. Refuse to answer
17. During the past 6 months, of all the times you had sex with another man, what proportion were in **bathhouses or sex clubs**? [SEXPLCE]
18. Never
19. Sometimes (<30% of the time)
20. Often (30-69% of the time)
21. Usually (70-95% of the time)
22. Always (>95% of the time)
23. Don’t know
24. Refuse to answer
25. During the past 6 months, of all the times you had sex with another man, what proportion were in **bars or nightclubs**? [SEXPLCF]
26. Never
27. Sometimes (<30% of the time)
28. Often (30-69% of the time)
29. Usually (70-95% of the time)
30. Always (>95% of the time)
31. Don’t know
32. Refuse to answer
33. During the past 6 months, of all the times you had sex with another man, what proportion were in **peep shows or sex shops**? [SEXPLCG]
34. Never
35. Sometimes (<30% of the time)
36. Often (30-69% of the time)
37. Usually (70-95% of the time)
38. Always (>95% of the time)
39. Don’t know
40. Refuse to answer
41. During the past 6 months, of all the times you had sex with another man, what proportion were in **a bareback scene**? [SEXPLCH]
42. Never
43. Sometimes (<30% of the time)
44. Often (30-69% of the time)
45. Usually (70-95% of the time)
46. Always (>95% of the time)
47. Don’t know
48. Refuse to answer
49. During the past 6 months, of all the times you had sex with another man, what proportion were in **a public place (park, restroom, or other public places)**?

[SEXPLCI]

1. Never
2. Sometimes (<30% of the time)
3. Often (30-69% of the time)
4. Usually (70-95% of the time)
5. Always (>95% of the time)
6. Don’t know
7. Refuse to answer
8. During the past 6 months, did you have sex with another man in **other places not mentioned previously**? [SEXPLCJ]
9. No  **🡪 go to question 43**
10. Yes
11. Don’t know
12. Refuse to answer

If yes,

- - 1. Please specify other places that you had sex with another man during the past 6 months? *(Use the keyboard to type or click letters on the screen)*  [SEXPLC1]

___________________________

- Don’t know
- Refuse to answer
  - 1. During the past 6 months, of all the times you had sex with another man, what proportion were in this place? [SEXPLC2]

1. Never
2. Sometimes (<30% of the time)
3. Often (30-69% of the time)
4. Usually (70-95% of the time)
5. Always (>95% of the time)
6. Don’t know
7. Refuse to answer
8. **During the past 6 months**, with how many different men have you had sex? *(oral or anal, including men you may have had sex with only once) If you don’t remember or don’t know exactly, please click the “Don’t know” button.* [NMPTN6M]

___________ (number of men, range 1- 999)

1. Don’t know (Don’t remember) **🡪 go to question 43.1**
2. Refuse to answer **🡪 go to question 43.1**

If Don’t know or Refuse to answer,

- 1. If you don’t remember or don’t know exactly, check one of the following that best fits your situation: [NMPTN6M1]

1. 1
2. 2 to 4
3. 5 to 9
4. 10 to 19
5. 20 to 49
6. 50 or more
7. Don’t know (Don’t remember)
8. Refuse to answer
9. **During the past 6 months**, how many of your male sex partners did you know were HIV-positive? [NMPTNPOS]

___________ (number of men, range 0- 999)

1. Don’t know
2. Refuse to answer
3. **During the past 6 months**, how many of your male sex partners told you they were HIV-negative and you had no reason to doubt it? [NMPTNNEG]

___________ (number of men, range 0- 999)

1. Don’t know
2. Refuse to answer
3. **During the past 6 months**, how many of your male sex partners never told you their HIV status? [NMPTNUNK]

___________ (number of men, range 0- 999)

1. Don’t know
2. Refuse to answer

**Sex with casual male partners**

*The following questions ask about your sexual behaviours with casual male partners. (A casual male partner is a man with whom you had sex only once (a “one night stand” or an encounter in a bathhouse, for example). Casual male partners do not include men to whom you gave or from whom you received money, drugs or other goods or services in exchange for sex).* [Info11]

1. **During the past 6 months**, have you had sex with a casual male partner (oral or anal)?

[CASPTN]

1. No **🡪 go to question 55**
2. Yes
3. Don’t know **🡪 go to question** **55**
4. Refuse to answer **🡪 go to question 55**
5. **During the past 6 months**, approximately how many **casual male** sexual partners have you had? *If you don’t remember or don’t know exactly, please click the “Don’t know” button.*  [NCASPTN]

___________ (number of men, range 1- 999)

1. Don’t know (Don’t remember) **🡪 go to question 48.1**
2. Refuse to answer **🡪 go to question 48.1**
   1. If you don’t remember exactly, check one of the following that fits to your situation: [NCASPTN1]
3. 1
4. 2 to 4
5. 5 to 9
6. 10 to 19
7. 20 to 49
8. 50 or more
9. Don’t know (Don’t remember)
10. Refuse to answer
11. Age distribution of casual male partners:
    1. **During the past 6 months**, how many of your casual male sexual partners were approximately the same age as you (i.e. within 3 years)? [AGECAS1]
12. None
13. Some of them
14. Most of them
15. All of them
16. Don’t know
17. Refuse to answer
    1. **During the past 6 months**, how many of your casual male sexual partners were more than 3 years younger than you? [AGECAS2]
18. None
19. Some of them
20. Most of them
21. All of them
22. Don’t know
23. Refuse to answer
    1. **During the past 6 months**, how many of your casual male sexual partners were more than 3 years older than you? [AGECAS3]
24. None
25. Some of them
26. Most of them
27. All of them
28. Don’t know
29. Refuse to answer

**Oral sex with casual male partners**

*The following questions ask about your oral sexual behaviors with casual male partners.*

[Info12]

1. **During the past 6 months**, have you had oral sex with a casual male partner where you sucked his cock? [CASSUCK]
2. No **🡪 go to question 51**
3. Yes
4. Don’t know **🡪 go to question** **51**
5. Refuse to answer **🡪 go to question 51**

If yes:

- 1. **During the past 6 months**, with how many casual male partners have you had oral sex? *(Number of men whose cock you sucked [be as precise as possible] )*

[CNSUCK]

___________ (number of men, range 1- 999)

1. Don’t know (Don’t remember)
2. Refuse to answer

- 1. **During the past 6 months**, have you had oral sex (sucking) **without a condom** with at least one casual male partner? ***(click all that apply)***  [CSKPTN]

1. who you knew at the time was HIV-positive?
2. who you knew at the time was HIV-negative?
3. whose HIV status you did not know at the time?
4. who you knew at the time had gonorrhea?
5. who you knew at the time had syphilis?
6. who you knew at the time had chlamydia?
7. who you knew at the time had genital or anal warts?
8. who you knew at the time had a sexually transmitted infection not mentioned above? **🡪 go to question 50.2a**
9. Don’t know
10. Refuse to answer
    - 1. Please specify the sexual infectious disease that your male partner had *(Use the keyboard to type or click letters on the screen)*  [CSKPTN1]

___________________________

- Don’t know
- Refuse to answer
  1. **During the past 6 months**, when you sucked the cock of your casual male partner, how often did you **use a condom**? [CSKCND1]

1. Never (0%)
2. Rarely (less than 25% of the time)
3. Sometimes (25-49% of the time)
4. Most of the time (50-74% of the time)
5. Almost every time (75-99% of the time)
6. All the time (100% of the time)
7. Don’t know
8. Refuse to answer
   1. **During the past 6 months**, when you sucked the cock of your casual male partner, how often did you **not** **use a condom but your partner did not cum in your mouth**? [CSKCND2]
9. Never (0%)
10. Rarely (less than 25% of the time)
11. Sometimes (25-49% of the time)
12. Most of the time (50-74% of the time)
13. Almost every time (75-99% of the time)
14. All the time (100% of the time)
15. Don’t know
16. Refuse to answer
    1. **During the past 6 months**, when you sucked the cock of your casual male partner, how often did you **not use a condom and your partner came in your mouth**? [CSKCND3]
17. Never (0%)
18. Rarely (less than 25% of the time)
19. Sometimes (25-49% of the time)
20. Most of the time (50-74% of the time)
21. Almost every time (75-99% of the time)
22. All the time (100% of the time)
23. Don’t know
24. Refuse to answer

**Anal sex with casual male partners**

*The following questions ask about your anal sexual behaviors with casual male partners.*

[Info13]

1. **During the past 6 months**, have you had anal sex with a casual male partner?[CASANAL]
2. No **🡪 go to question 55**
3. Yes
4. Don’t know **🡪 go to question** **55**
5. Refuse to answer **🡪 go to question 55**

If yes:

- 1. **During the past 6 months**, with how many casual male partners have you had anal sex? *(please be as precise as possible)* [CNANAL]

___________ (number of men, range 1- 999)

1. Don’t know
2. Refuse to answer
   1. Did you or your partner use a condom **the last time you had anal sex** with a casual male partner? [CANLCNDL]
3. No
4. Yes
5. Don’t know
6. Refuse to answer
7. **During the past 6 months**, have you had unprotected anal sex (i.e. without a condom) with at least one casual male partner? ***(click all that apply)*** [CANLPTN]
8. who you knew at the time was HIV-positive?
9. who you knew at the time was HIV-negative?
10. whose HIV status you did not know at the time?
11. who you knew at the time had gonorrhea?
12. who you knew at the time had syphilis?
13. who you knew at the time had chlamydia?
14. who you knew at the time had genital or anal warts?
15. who you knew at the time had a sexually transmitted infection not mentioned above?

**🡪 go to question 52.1**

- 1. Don’t know

1. Refuse to answer
   1. Please specify the sexual infectious disease that your male partner had *(Use the keyboard to type or click letters on the screen)*  [CANLPTN1]

___________________________

- Don’t know
- Refuse to answer

1. **During the past 6 months**, did you fuck your casual partners (anal sex)? [CASINS]
2. No **🡪 go to question 54**
3. Yes
4. Don’t know **🡪 go to question** **54**
5. Refuse to answer **🡪 go to question 54**

If yes,

- 1. **During the past 6 months**, how often did you use a condom when you fucked your casual partner? [CINSCND1]

1. Never (0%)
2. Rarely (less than 25% of the time)
3. Sometimes (25-49% of the time)
4. Most of the time (50-74% of the time)
5. Almost every time (75-99% of the time)
6. All the time (100% of the time)
7. Don’t know
8. Refuse to answer
   1. **During the past 6 months**, have you ever partially or fully inserted your cock into a casual male partner’s ass before putting a condom on? [CINSCND2]
9. No
10. Yes, once
11. Yes, more than once
12. Don’t know
13. Refuse to answer
    1. **During the past 6 months**, while fucking, have you ever taken the condom off and then continued to fuck your casual partner? [CINSCND3]
14. No
15. Yes, once
16. Yes, more than once
17. Don’t know
18. Refuse to answer
19. **During the past 6 months**, did your casual partners fuck you (anal sex)? [CASREC]
20. No **🡪 go to question 55**
21. Yes
22. Don’t know **🡪 go to question** **55**
23. Refuse to answer **🡪 go to question 55**

If yes,

- 1. **During the past 6 months**, how often did your casual male partner use a condom when he fucked you? [CRECCND1]

1. Never (0%)
2. Rarely (less than 25% of the time)
3. Sometimes (25-49% of the time)
4. Most of the time (50-74% of the time)
5. Almost every time (75-99% of the time)
6. All the time (100% of the time)
7. Don’t know
8. Refuse to answer
   1. **During the past 6 months**, has a casual male partner ever partially or fully inserted his cock into your ass before putting a condom on? [CRECCND2]
9. No
10. Yes, once
11. Yes, more than once
12. Don’t know
13. Refuse to answer
    1. **During the past 6 months**, while being fucked, has a casual male partner ever taken the condom off and then continued to fuck you? [CRECCND3]
14. No
15. Yes, once
16. Yes, more than once
17. Don’t know
18. Refuse to answer

**Sex with regular male partners**

*The following questions ask about your sexual behaviours with regular male partners. (A regular male partner is a man with whom you had sex at least twice. It can be a life partner, a boyfriend, a “fuck-friend”, a lover, etc. Regular male partners do not include men to whom you gave or from whom you received money, drugs or other goods or services in exchange for sex)*

[Info14]

1. **During the past 6 months**, have you had sex with a regular male partner (oral or anal)?

[REGPTN]

1. No **🡪 go to question 61**
2. Yes
3. Don’t know **🡪 go to question** **61**
4. Refuse to answer **🡪 go to question 61**
5. **During the past 6 months**, approximately how many **regular male** sexual partners have you had? *If you don’t remember or don’t know exactly, please click the “Don’t know” button.* [NREGPTN]

___________ (number of men, range 1- 999)

1. Don’t know (Don’t remember) **🡪 go to question 56.1**
2. Refuse to answer **🡪 go to question 56.1**
   1. If you don’t remember exactly, check one of the following that fits to your situation: [NREGPTN1]
3. 1
4. 2 to 4
5. 5 to 9
6. 10 to 19
7. 20 to 49
8. 50 or more
9. Don’t know (Don’t remember)
10. Refuse to answer
11. Age distribution of regular male partners:
    1. **During the past 6 months**, how many of your regular male sexual partners were approximately the same age as you (i.e. within 3 years)? [AGEREG1]
12. None
13. Some of them
14. Most of them
15. All of them
16. Don’t know
17. Refuse to answer
    1. **During the past 6 months**, how many of your regular male sexual partners were more than 3 years younger than you? [AGEREG2]
18. None
19. Some of them
20. Most of them
21. All of them
22. Don’t know
23. Refuse to answer
    1. **During the past 6 months**, how many of your regular male sexual partners were more than 3 years older than you? [AGEREG3]
24. None
25. Some of them
26. Most of them
27. All of them
28. Don’t know
29. Refuse to answer

**Sex with regular HIV-positive male partners**

*The following questions ask about your sexual behaviors with regular HIV-positive male partners.*  [Info15]

1. **During the past 6 months**, have you had sex (oral or anal) with a regular HIV-positive male partner? [REGPOS]
2. No **🡪 go to question 59**
3. Yes
4. Don’t know **🡪 go to question 59**
5. Refuse to answer **🡪 go to question 59**

If yes,

**Oral sex with regular HIV-positive male partners**

- 1. **During the past 6 months**, have you had oral sex with a regular HIV-positive male partner where you sucked his cock? [POSSUCK]

1. No **🡪 go to question 58.2**
2. Yes
3. Don’t know **🡪 go to question 58.2**
4. Refuse to answer **🡪 go to question 58.2**

If yes:

- - 1. **During the past 6 months**, with how many **regular HIV-positive** male partners have you had oral sex? *(Number of men whose cock you sucked [please be as precise as possible])* [PNSUCK]

___________ (number of men, range 1- 999)

1. Don’t know (Don’t remember)
2. Refuse to answer
   - 1. **During the past 6 months**, when you sucked the cock of your regular HIV-positive male partner, how often did you **use a condom**?

[PSKCND1]

1. Never (0%)
2. Rarely (less than 25% of the time)
3. Sometimes (25-49% of the time)
4. Most of the time (50-74% of the time)
5. Almost every time (75-99% of the time)
6. All the time (100% of the time)
7. Don’t know
8. Refuse to answer
   - 1. **During the past 6 months**, when you sucked the cock of your regular HIV-positive male partner, how often did you **not use a condom but your partner did not cum in your mouth**? [PSKCND2]
9. Never (0%)
10. Rarely (less than 25% of the time)
11. Sometimes (25-49% of the time)
12. Most of the time (50-74% of the time)
13. Almost every time (75-99% of the time)
14. All the time (100% of the time)
15. Don’t know
16. Refuse to answer
    - 1. **During the past 6 months**, when you sucked the cock of your regular HIV-positive male partner, how often did you **not use a condom and your partner came in your mouth**? [PSKCND3]
17. Never (0%)
18. Rarely (less than 25% of the time)
19. Sometimes (25-49% of the time)
20. Most of the time (50-74% of the time)
21. Almost every time (75-99% of the time)
22. All the time (100% of the time)
23. Don’t know
24. Refuse to answer

**Anal sex with regular HIV-positive male partners**

- 1. **During the past 6 months**, have you had anal sex with a regular HIV-positive male partner? [POSANAL]

1. No **🡪 go to question 59**
2. Yes
3. Don’t know **🡪 go to question 59**
4. Refuse to answer **🡪 go to question 59**

If yes:

- - 1. Did you or your partner use a condom **the last time you had anal sex** with a regular HIV-positive male partner? [PANLCNDL]

1. No
2. Yes
3. Don’t know
4. Refuse to answer
   - 1. **During the past 6 month**, did you fuck these regular HIV-positive male partners (anal sex)? [POSINS]
5. No **🡪 go to question 58.2c**
6. Yes
7. Don’t know **🡪 go to question 58.2c**
8. Refuse to answer **🡪 go to question 58.2c**

If yes,

- - - 1. **During the past 6 months**, how often did you use a condom when you fucked your regular HIV-positive male partner?

[PINSCND1]

1. Never (0%)
2. Rarely (less than 25% of the time)
3. Sometimes (25-49% of the time)
4. Most of the time (50-74% of the time)
5. Almost every time (75-99% of the time)
6. All the time (100% of the time)
7. Don’t know
8. Refuse to answer
   - - 1. **During the past 6 months**, have you ever partially or fully inserted your cock into a regular HIV-positive male partner’s ass before putting a condom on? [PINSCND2]
9. No
10. Yes, once
11. Yes, more than once
12. Don’t know
13. Refuse to answer
    - - 1. **During the past 6 months**, while fucking, have you ever taken the condom off and then continued to fuck your regular HIV-positive partner? [PINSCND3]
14. No
15. Yes, once
16. Yes, more than once
17. Don’t know
18. Refuse to answer
    - 1. **During the past 6 months**, did these regular HIV-positive male partners fuck you (anal sex)? [POSREC]
19. No **🡪 go to question 59**
20. Yes
21. Don’t know **🡪 go to question 59**
22. Refuse to answer **🡪 go to question 59**

If yes,

- - - 1. **During the past 6 months**, how often did your regular HIV-positive male partner use a condom when he fucked you?

[PRECCND1]

1. Never (0%)
2. Rarely (less than 25% of the time)
3. Sometimes (25-49% of the time)
4. Most of the time (50-74% of the time)
5. Almost every time (75-99% of the time)
6. All the time (100% of the time)
7. Don’t know
8. Refuse to answer
   - - 1. **During the past 6 months**, has a regular HIV-positive male partner ever partially or fully inserted his cock into your ass before putting a condom on? [PRECCND2]
9. No
10. Yes, once
11. Yes, more than once
12. Don’t know
13. Refuse to answer
    - - 1. **During the past 6 months**, while being fucked, has a regular HIV-positive male partner ever taken the condom off and then continued to fuck you? [PRECCND3]
14. No
15. Yes, once
16. Yes, more than once
17. Don’t know
18. Refuse to answer

**Sex with regular male partners whose HIV status was unknown to you**

*The following questions ask about your sexual behaviors with regular male partners* *whose HIV status was unknown to you.*  [Info16]

1. **During the past 6 months**, have you had sex (oral or anal) with regular male partner whose HIV status was unknown to you? [REGUNK]
2. No **🡪 go to question 60**
3. Yes
4. Don’t know **🡪 go to question 60**
5. Refuse to answer **🡪 go to question 60**

If yes,

**Oral sex with regular male partners whose HIV status was unknown to you**

- 1. **During the past 6 months**, have you sucked the cock (oral sex) of a regular male partner whose HIV status was unknown to you? [UNKSUCK]

1. No **🡪 go to question 59.2**
2. Yes
3. Don’t know **🡪 go to question 59.2**
4. Refuse to answer **🡪 go to question 59.2**

If yes:

- - 1. **During the past 6 months**, with how many regular male partners whose HIV status was unknown to you have you had oral sex? *(Number of men whose cock you sucked [please be as precise as possible])*

[UNSUCK]

___________ (number of men, range 1- 999)

1. Don’t know (Don’t remember)
2. Refuse to answer
   - 1. **During the past 6 months**, when you sucked the cock of your regular male partner whose HIV status was unknown to you, how often did you suck **with a condom**? [USKCND1]
3. Never (0%)
4. Rarely (less than 25% of the time)
5. Sometimes (25-49% of the time)
6. Most of the time (50-74% of the time)
7. Almost every time (75-99% of the time)
8. All the time (100% of the time)
9. Don’t know
10. Refuse to answer
    - 1. **During the past 6 months**, when you sucked the cock of your regular male partner whose HIV status was unknown to you, how often did you **not use** **a condom but your partner did not cum in your mouth**?

[USKCND2]

1. Never (0%)
2. Rarely (less than 25% of the time)
3. Sometimes (25-49% of the time)
4. Most of the time (50-74% of the time)
5. Almost every time (75-99% of the time)
6. All the time (100% of the time)
7. Don’t know
8. Refuse to answer
   - 1. **During the past 6 months**, when you sucked the cock of your regular male partner whose HIV status was unknown to you, how often did you **not use a condom and your partner came in your mouth**?

[USKCND3]

1. Never (0%)
2. Rarely (less than 25% of the time)
3. Sometimes (25-49% of the time)
4. Most of the time (50-74% of the time)
5. Almost every time (75-99% of the time)
6. All the time (100% of the time)
7. Don’t know
8. Refuse to answer

**Anal sex with regular male partners whose HIV status was unknown to you**

- 1. **During the past 6 months**, have you had anal sex with a regular male partner whose HIV status was unknown to you? [UNKANAL]

1. No **🡪 go to question 60**
2. Yes
3. Don’t know **🡪 go to question 60**
4. Refuse to answer **🡪 go to question 60**

If yes:

- - 1. Did you or your partner use a condom **the last time you had anal sex** with a regular male partner whose HIV status was unknown to you?

[UANLCNDL]

1. No
2. Yes
3. Don’t know
4. Refuse to answer
   - 1. **During the past 6 months**, did you fuck these regular male partners whose HIV status was unknown to you (anal sex)? [UNKINS]
5. No **🡪 go to question 59.2c**
6. Yes
7. Don’t know **🡪 go to question 59.2c**
8. Refuse to answer **🡪 go to question 59.2c**

If yes,

- - - 1. **During the past 6 months**, how often did you use a condom when you fucked your regular male partner whose HIV status was unknown to you? [UINSCND1]

1. Never (0%)
2. Rarely (less than 25% of the time)
3. Sometimes (25-49% of the time)
4. Most of the time (50-74% of the time)
5. Almost every time (75-99% of the time)
6. All the time (100% of the time)
7. Don’t know
8. Refuse to answer
   - - 1. **During the past 6 months**, have you ever partially or fully inserted your cock into the ass of a regular male partner whose HIV status was unknown to you before putting a condom on?

[UINSCND2]

1. No
2. Yes, once
3. Yes, more than once
4. Don’t know
5. Refuse to answer
   - - 1. **During the past 6 months**, while fucking, have you ever taken the condom off and then continued to fuck your regular male partner whose HIV status was unknown to you? [UINSCND3]
6. No
7. Yes, once
8. Yes, more than once
9. Don’t know
10. Refuse to answer
    - 1. **During the past 6 months**, did these regular male partners whose HIV status was unknown to you fuck you (anal sex)? [UNKREC]
11. No **🡪 go to question 60**
12. Yes
13. Don’t know **🡪 go to question 60**
14. Refuse to answer **🡪 go to question 60**

If yes,

- - - 1. **During the past 6 months**, how often did your regular male partner whose HIV status was unknown to you use a condom when he fucked you? [URECCND1]

1. Never (0%)
2. Rarely (less than 25% of the time)
3. Sometimes (25-49% of the time)
4. Most of the time (50-74% of the time)
5. Almost every time (75-99% of the time)
6. All the time (100% of the time)
7. Don’t know
8. Refuse to answer
   - - 1. **During the past 6 months**, has a regular male partner whose HIV status was unknown to you ever partially or fully inserted his cock into your ass before putting a condom on? [URECCND2]
9. No
10. Yes, once
11. Yes, more than once
12. Don’t know
13. Refuse to answer
    - - 1. **During the past 6 months**, while being fucked, has a regular male partner whose HIV status was unknown to you ever taken the condom off and then continued to fuck you? [URECCND3]
14. No
15. Yes, once
16. Yes, more than once
17. Don’t know
18. Refuse to answer

**Sex with regular HIV-negative male partners**

*The following questions ask about your sexual behaviors with regular HIV-negative male partners.*  [Info17]

1. **During the past 6 months**, have you had sex (oral or anal) with regular HIV-negative male partners? [REGNEG]
2. No **🡪 go to question 61**
3. Yes
4. Don’t know **🡪 go to question 61**
5. Refuse to answer **🡪 go to question 61**

If yes,

**Oral sex with regular HIV-negative male partners**

- 1. **During the past 6 months**, have you had oral sex with a regular HIV-negative male partner where you sucked his cock? [NEGSUCK]

1. No **🡪 go to question 60.2**
2. Yes
3. Don’t know **🡪 go to question 60.2**
4. Refuse to answer **🡪 go to question 60.2**

If yes:

- - 1. **During the past 6 months**, with how many **regular HIV-negative** male partners have you had oral sex? *(Number of men whose cock you sucked [please be as precise as possible])*  [NNSUCK]

___________ (number of men, range 1- 999)

1. Don’t know (Don’t remember)
2. Refuse to answer
   - 1. **During the past 6 months**, when you sucked the cock of your regular HIV-negative male partner, how often did you **use a condom**?

[NSKCND1]

1. Never (0%)
2. Rarely (less than 25% of the time)
3. Sometimes (25-49% of the time)
4. Most of the time (50-74% of the time)
5. Almost every time (75-99% of the time)
6. All the time (100% of the time)
7. Don’t know
8. Refuse to answer
   - 1. **During the past 6 months**, when you sucked the cock of your regular HIV-negative male partner, how often did you **not use a condom but your partner did not cum in your mouth**? [NSKCND2]
9. Never (0%)
10. Rarely (less than 25% of the time)
11. Sometimes (25-49% of the time)
12. Most of the time (50-74% of the time)
13. Almost every time (75-99% of the time)
14. All the time (100% of the time)
15. Don’t know
16. Refuse to answer
    - 1. **During the past 6 months**, when you sucked the cock of your regular HIV-negative male partner, how often did you **not use a condom and your partner came in your mouth**? [NSKCND3]
17. Never (0%)
18. Rarely (less than 25% of the time)
19. Sometimes (25-49% of the time)
20. Most of the time (50-74% of the time)
21. Almost every time (75-99% of the time)
22. All the time (100% of the time)
23. Don’t know
24. Refuse to answer

**Anal sex with regular HIV-negative male partners**

- 1. **During the past 6 months**, have you had anal sex with a regular HIV-negative male partner? [NEGANAL]

1. No **🡪 go to question 61**
2. Yes
3. Don’t know **🡪 go to question 61**
4. Refuse to answer **🡪 go to question 61**

If yes:

- - 1. Did you or your partner use a condom **the last time you had anal sex** with a regular HIV-negative male partner? [NANLCNDL]

1. No
2. Yes
3. Don’t know
4. Refuse to answer
   - 1. **During the past 6 months**, did you fuck these regular HIV-negative male partners (anal sex)? [NEGINS]
5. No **🡪 go to question 60.2c**
6. Yes
7. Don’t know **🡪 go to question 60.2c**
8. Refuse to answer **🡪 go to question 60.2c**

If yes,

- - - 1. **During the past 6 months**, how often did you use a condom when you fucked your regular HIV-negative male partner?

[NINSCND1]

1. Never (0%)
2. Rarely (less than 25% of the time)
3. Sometimes (25-49% of the time)
4. Most of the time (50-74% of the time)
5. Almost every time (75-99% of the time)
6. All the time (100% of the time)
7. Don’t know
8. Refuse to answer
   - - 1. **During the past 6 months**, have you ever partially or fully inserted your cock into a regular HIV-negative male partner’s ass before putting a condom on? [NINSCND2]
9. No
10. Yes, once
11. Yes, more than once
12. Don’t know
13. Refuse to answer
    - - 1. **During the past 6 months**, while fucking, have you ever taken the condom off and then continued to fuck your regular HIV-negative partner? [NINSCND3]
14. No
15. Yes, once
16. Yes, more than once
17. Don’t know
18. Refuse to answer
    - 1. **During the past 6 months**, did these regular HIV-negative male partners fuck you (anal sex)? [NEGREC]
19. No **🡪 go to question 61**
20. Yes
21. Don’t know **🡪 go to question 61**
22. Refuse to answer **🡪 go to question 61**

If yes,

- - - 1. **During the past 6 months**, how often did your regular HIV-negative male partner use a condom when he fucked you?

[NRECCND1]

1. Never (0%)
2. Rarely (less than 25% of the time)
3. Sometimes (25-49% of the time)
4. Most of the time (50-74% of the time)
5. Almost every time (75-99% of the time)
6. All the time (100% of the time)
7. Don’t know
8. Refuse to answer
   - - 1. **During the past 6 months**, has a regular HIV-negative male partner ever partially or fully inserted his cock into your ass before putting a condom on? [NRECCND2]
9. No
10. Yes, once
11. Yes, more than once
12. Don’t know
13. Refuse to answer
    - - 1. **During the past 6 months**, while being fucked, has a regular HIV-negative male partner ever taken the condom off and then continued to fuck you? [NRECCND3]
14. No
15. Yes, once
16. Yes, more than once
17. Don’t know
18. Refuse to answer

**Sex in exchange for money, drugs or goods**

*The next question asks you about sex in exchange for money, drugs or other goods or services.*

[Info18]

1. **During the past 6 months**, have you given or received the following things in exchange for sex (oral or anal)? ***(click all that apply)*** [SEXWK]
2. Have you given money to another man for sex?
3. Have you given drugs to another man for sex?
4. Have you given other goods or services to another man in exchange for sex (e.g. room, meal, etc.)?
5. Have you received money from another man for sex?
6. Have you received drugs from another man for sex?
7. Have you received other goods or services from another man in exchange for sex (e.g. room, meal, etc.)?
8. None of the above
   1. Don’t know
9. Refuse to answer

**Attitude and knowledge on condom use**

*The following questions ask about your attitude and knowledge on condom use.*  [Info19]

1. We would like to know about your opinions regarding condoms. What do you think about the following statements? [Info20]
2. Condoms can effectively prevent sexually transmitted infections (STIs).

[OPINCNDA]

1. Strongly agree
2. Agree
3. Neutral (neither agree nor disagree)
4. Disagree
5. Strongly disagree
6. Don’t know
7. Refuse to answer
8. Condoms can effectively prevent HIV/AIDS. [OPINCNDB]
9. Strongly agree
10. Agree
11. Neutral (neither agree nor disagree)
12. Disagree
13. Strongly disagree
14. Don’t know
15. Refuse to answer
16. I can usually persuade my partner to use a condom even if he does not really want to. [OPINCNDC]
17. Strongly agree
18. Agree
19. Neutral (neither agree nor disagree)
20. Disagree
21. Strongly disagree
22. Don’t know
23. Refuse to answer
24. I will refuse to have sex if my partner does not want to use a condom and I feel at risk for STI/HIV. [OPINCNDD]
25. Strongly agree
26. Agree
27. Neutral (neither agree nor disagree)
28. Disagree
29. Strongly disagree
30. Don’t know
31. Refuse to answer
32. I know where to get condoms. [OPINCNDE]
33. Strongly agree
34. Agree
35. Neutral (neither agree nor disagree)
36. Disagree
37. Strongly disagree
38. Don’t know
39. Refuse to answer
40. You can use cooking oil or Vaseline for lubrication when using a condom.

[OPINCNDF]

1. Strongly agree
2. Agree
3. Neutral (neither agree nor disagree)
4. Disagree
5. Strongly disagree
6. Don’t know
7. Refuse to answer
8. Some space should be left at the tip of the condom to allow for sperm.

[OPINCNDG]

1. Strongly agree
2. Agree
3. Neutral (neither agree nor disagree)
4. Disagree
5. Strongly disagree
6. Don’t know
7. Refuse to answer
8. A condom should be unrolled before putting it on the penis. [OPINCNDH]
9. Strongly agree
10. Agree
11. Neutral (neither agree nor disagree)
12. Disagree
13. Strongly disagree
14. Don’t know
15. Refuse to answer
16. Condoms often break. [OPINCNDI]
17. Strongly agree
18. Agree
19. Neutral (neither agree nor disagree)
20. Disagree
21. Strongly disagree
22. Don’t know
23. Refuse to answer
24. If someone is willing to have sex without a condom, I just assume that he is HIV negative. [OPINCNDJ]
25. Strongly agree
26. Agree
27. Neutral (neither agree nor disagree)
28. Disagree
29. Strongly disagree
30. Don’t know
31. Refuse to answer
32. If someone is willing to have sex without a condom, I just assume that he is HIV positive. [OPINCNDK]
33. Strongly agree
34. Agree
35. Neutral (neither agree nor disagree)
36. Disagree
37. Strongly disagree
38. Don’t know
39. Refuse to answer
40. Sometimes I feel depressed about not having a relationship and give in when it comes to sex even if it is without a condom. [OPINCNDL]
41. Strongly agree
42. Agree
43. Neutral (neither agree nor disagree)
44. Disagree
45. Strongly disagree
46. Don’t know
47. Refuse to answer
48. I respect whatever the guy wants regardless of whether he’s positive or negative. If he wants to use a condom, that’s fine, and if he doesn’t, that’s fine too.

[OPINCNDM]

1. Strongly agree
2. Agree
3. Neutral (neither agree nor disagree)
4. Disagree
5. Strongly disagree
6. Don’t know
7. Refuse to answer
8. It is not up to me to take responsibility for guys I meet for sex. They are adults who can make their own decisions around risk. [OPINCNDN]
9. Strongly agree
10. Agree
11. Neutral (neither agree nor disagree)
12. Disagree
13. Strongly disagree
14. Don’t know
15. Refuse to answer
16. Most guys fuck with condoms when they do not know the other guy. [OPINCNDO]
17. Strongly agree
18. Agree
19. Neutral (neither agree nor disagree)
20. Disagree
21. Strongly disagree
22. Don’t know
23. Refuse to answer
24. A lot of guys I have sex with have no desire to use condoms. [OPINCNDP]
25. Strongly agree
26. Agree
27. Neutral (neither agree nor disagree)
28. Disagree
29. Strongly disagree
30. Don’t know
31. Refuse to answer
32. If I lose my erection with a condom on, I prefer to have sex without it.

[OPINCNDQ]

1. Strongly agree
2. Agree
3. Neutral (neither agree nor disagree)
4. Disagree
5. Strongly disagree
6. Don’t know
7. Refuse to answer
8. Reasons for not always using a condom

**[If never had sex with a man (SEXMEN ^=1) or no sex with a man during the past 6 months (SEXM6M ^=1) or no regular partner during the past 6 months (REGPTN ^=1) 🡪 go to question 63.2]**

- 1. **During the past 6 months**, did you always use a condom when you had sex with your regular male partner? [ALWSCNDR]

1. No
2. Yes **🡪 go to question 63.2**
3. Don’t know
4. Refuse to answer
   - 1. Which of these following reasons are why you did not always use a condom when you had sex with your regular male partner? ***(next screen has additional reasons) (click all reasons that apply)*** [NOCDR1]
5. You don’t like using condoms
6. Your partner doesn’t like using condoms or he refuses to use condoms
7. You thought your partner was at low risk for getting HIV or AIDS
8. You were in a mutually faithful sexual relationship
9. You feared you would lose the relationship if you insisted on using a condom **🡪 go to question 63.1d**
10. You didn’t think you could get the AIDS virus or pass the AIDS virus on to others
11. You knew you and your partner had the same HIV status (both HIV-negative or both HIV-positive)
12. You knew you were HIV-negative
13. You knew your partner was HIV-negative
    1. Don’t know **🡪 go to question 63.2**
14. Refuse to answer **🡪 go to question 63.2**
    - 1. Which of these following reasons are why you did not always use a condom when you had sex with your regular male partner? ***(continued) (click all reasons that apply)*** [NOCDR2]
15. No condom was available when you were having sex
16. You were too embarrassed to get condoms
17. You could not talk about using a condom with your partner
18. You were in the heat of the moment
19. Condoms were not effective, they often slipped, broke or leaked during sex
20. You were too drunk or you were using drugs
21. You were forced to have sex against your will
22. You were afraid your partner would accuse you of having sex with another person or other people
23. You couldn’t afford to buy enough condoms to use them every time you had sex
24. Other **🡪 go to question 63.1c**
    - 1. Please specify the other reasons you did not always use a condom when you had sex with your regular male partner *(Use the keyboard to type or click letters on the screen)*  [NOCDR3]

__________________________

- - - - Don’t know
      - Refuse to answer
    1. You indicated that did not always use a condom when you had sex with your regular male partner because you feared you would lose the relationship. Your regular partner is: [NOCDR4]

1. About the same age as you (i.e. within 3 years)
2. Younger than you (by more than 3 years)
3. Older than you (by more than years)
4. Don’t know
5. Refuse to answer

**[If never had sex with a man (SEXMEN ^=1) or no sex with a man during the past 6 months (SEXM6M ^=1) or (no casual partner during the past 6 months (CASPTN ^=1) 🡪 go to question 63.3]**

- 1. **During the past 6 months**, did you always use a condom when you had sex with a casual male partner? [ALWSCNDC]
  2. No
  3. Yes **🡪 go to question 63.3**

1. Don’t know
2. Refuse to answer
   - 1. Which of these following reasons are why you did not always use a condom when you had sex with a casual male partner? ***(next screen has additional reasons) (click all reasons that apply)***

[NOCDC1]

1. You don’t like using condoms
2. Your partner doesn’t like using condoms or he refuses to use condoms
3. You were afraid your partner would not want to have sex with you if you insisted on using a condom
4. You thought your partner was at low risk for getting HIV or AIDS
5. You didn’t think you could get the AIDS virus or pass the AIDS virus on to others
6. You knew you and your partner had the same HIV status
7. You knew you were HIV-negative
8. You knew your partner was HIV-negative
   1. Don’t know **🡪 go to question 63.3**
9. Refuse to answer **🡪 go to question 63.3**
   - 1. Which of these following reasons are why you did not always use a condom when you had sex with a casual male partner? ***(continued) (click all reasons that apply)*** [NOCDC2]
10. No condom was available when you were having sex
11. You were too embarrassed to get condoms
12. You could not talk about using a condom with your partner
13. You were in the heat of the moment
14. Condoms were not effective, they often slipped, broke or leaked during sex
15. You were too drunk or you were using drugs
16. You were forced to have sex against your will
17. You couldn’t afford to buy enough condoms to use them every time you had sex
18. Other **🡪 go to question 63.2c**
    - 1. Please specify the other reasons you did not always use a condom when you had sex with your casual male partner *(Use the keyboard to type or click letters on the screen)*  [NOCDC3]

__________________________

- - - - Don’t know
      - Refuse to answer

**[If never had sex with a man (SEXMEN ^=1) or no sex with a man during the past 6 months (SEXM6M ^=1) or not Sex Worker (SEXWK = 0 or SEXWK=REF or SEXWK=DK or (SEXWK = 1 and SEXWKG = 1)) 🡪 go to question 64]**

- 1. **During the past 6 months**, did you always use a condom when you had sex with a male sex worker or a male client? [ALWSCNDS]
  2. No
  3. Yes **🡪 go to question 64**

1. Don’t know
2. Refuse to answer
   - 1. Which of these following reasons are why you did not always use a condom when you had sex with a male sex worker or a male client? ***(next screen has additional reasons) (click all reasons that apply)***

[NOCDSW1]

1. You don’t like using condoms
2. Your partner doesn’t like using condoms or he refuses to use condoms
3. You were afraid your partner would not want to have sex with you if you insisted on using a condom
4. You thought your partner was at low risk for getting HIV or AIDS
5. You didn’t think you could get the AIDS virus or pass the AIDS virus on to others
6. You knew you and your partner had the same HIV status
7. You knew you were HIV-negative
8. You knew your partner was HIV-negative
   1. Don’t know **🡪 go to question 64**
9. Refuse to answer **🡪 go to question 64**
   - 1. Which of these following reasons are why you did not always use a condom when you had sex with a male sex worker or a male client? ***(continued) (click all reasons that apply)*** [NOCDSW2]
10. No condom was available when you were having sex
11. You were too embarrassed to get condoms
12. You could not talk about using a condom with your partner
13. You were in the heat of the moment
14. Condoms were not effective, they often slipped, broke or leaked during sex
15. You were too drunk or you were using drugs
16. You were forced to have sex against your will
17. You couldn’t afford to buy enough condoms to use them every time you had sex
18. Other **🡪 go to question 63.2c**
    - 1. Please specify the other reasons you did not always use a condom when you had sex with a male sex worker or a male client *(Use the keyboard to type or click letters on the screen)*  [NOCDSW3]

__________________________

- - - - Don’t know
      - Refuse to answer

1. **During the past 6 months**, did you experience any situation that put you at high risk for HIV or a sexually transmitted disease? [HRISK]
2. No **🡪 go to question 65**
3. Yes
4. Don’t know **🡪 go to question 65**
5. Refuse to answer **🡪 go to question 65**

If **yes,**

- 1. Please describe in detail what happened during this situation (when, where, how, why …) *(For example, last year at some place, you had sex without a condom with a man who later you knew was HIV-infected) (Use the keyboard to type or click letters on the screen)*  [HRISK1]

________________________________________________________________________________________________________________________________________________________________________________________________________________________________________________________________________

- Don’t know
- Refuse to answer

**SECTION 4: STI, HEPATITIS AND HIV TESTING, DIAGNOSIS AND TREATMENT**

*The following questions ask about the tests for sexually transmitted infections, hepatitis and HIV you may have had when seeing a health professional.* [Info21]

1. Diagnosed or treated diseases
2. Have you ever had gonorrhea? [DISA]
   1. No
   2. Yes **🡪 go to question 65A.1**
3. Don’t know
4. Refuse to answer
5. Please specify the first time you had gonorrhea [DISA1]

______ (year) ______ (month)

1. Don’t know

**[Check: should be later than year of birth, as well as, before date of interview 🡪 loop back to question 65A.1]**

**Message:**

The year of first time you had gonorrhea should be later than the year of your birth, as well as, before today. Please correct it.

1. Have you ever had chlamydia? [DISB]
2. No
3. Yes **🡪 go to question 65B.1**
4. Don’t know
5. Refuse to answer
6. Please specify the first time you had chlamydia [DISB1]

______ (year) ______ (month)

1. Don’t know

**[Check: should be later than year of birth, as well as, before date of interview 🡪 loop back to question 65B.1]**

**Message:**

The year of first time you had chlamydia should be later than the year of your birth, as well as, before today. Please correct it.

1. Have you ever had syphilis? [DISC]
2. No
3. Yes **🡪 go to question 65C.1**
4. Don’t know
5. Refuse to answer
6. Please specify the first time you had syphilis [DISC1]

______ (year) ______ (month)

1. Don’t know

**[Check: should be later than year of birth, as well as, before date of interview 🡪 loop back to question 65C.1]**

**Message:**

The year of first time you had syphilis should be later than the year of your birth, as well as, before today. Please correct it.

1. Have you ever had genital or anal warts? [DISD]
2. No
3. Yes **🡪 go to question 65D.1**
4. Don’t know
5. Refuse to answer
6. Please specify the first time you had genital or anal warts [DISD1]

______ (year) ______ (month)

1. Don’t know

**[Check: should be later than year of birth, as well as, before date of interview 🡪 loop back to question 65D.1]**

**Message:**

The year of first time you had genital or anal warts should be later than the year of your birth, as well as, before today. Please correct it.

1. Have you ever had genital herpes? [DISE]
2. No
3. Yes **🡪 go to question 65E.1**
4. Don’t know
5. Refuse to answer
6. Please specify the first time you had genital herpes [DISE1]

______ (year) ______ (month)

- - 1. Don’t know

**[Check: should be later than year of birth, as well as, before date of interview 🡪 loop back to question 65E.1]**

**Message:**

The year of first time you had genital herpes should be later than the year of your birth, as well as, before today. Please correct it.

1. Have you ever had genital ulcers? [DISF]
2. No
3. Yes **🡪 go to question 65F.1**
4. Don’t know
5. Refuse to answer
6. Please specify the first time you had genital ulcers [DISF1]

______ (year) ______ (month)

1. Don’t know

**[Check: should be later than year of birth, as well as, before date of interview 🡪 loop back to question 65F.1]**

**Message:**

The year of first time you had genital ulcers should be later than the year of your birth, as well as, before today. Please correct it.

1. Have you ever had lymphogranuloma venereum (LGV) infection? [DISG]
2. No
3. Yes **🡪 go to question 65G.1**
4. Don’t know
5. Refuse to answer
6. Please specify the first time you had lymphogranuloma venereum (LGV) infection [DISG1]

______ (year) ______ (month)

1. Don’t know

**[Check: should be later than year of birth, as well as, before date of interview 🡪 loop back to question 65G.1]**

**Message:**

The year of first time you had lymphogranuloma venereum (LGV) infection should be later than the year of your birth, as well as, before today. Please correct it.

1. Have you ever had a sexually transmitted infection (STI)/sexually transmitted disease (STD) but you have forgotten the name? [DISH]
2. No
3. Yes **🡪 go to question 65H.1**
4. Don’t know
5. Refuse to answer
6. Please specify the first time you had an STI/STD that you have forgotten the name [DISH1]

______ (year) ______ (month)

1. Don’t know

**[Check: should be later than year of birth, as well as, before date of interview 🡪 loop back to question 65H.1]**

**Message:**

The year of first time you had an STI/STD that you have forgotten the name should be later than the year of your birth, as well as, before today. Please correct it.

1. Have you ever had any other sexually transmitted infection (STI)/sexually transmitted disease (STD) not mentioned in the previous questions? [DISI]
2. No
3. Yes **🡪 go to question 65I.1**
4. Don’t know
5. Refuse to answer
6. Please specify the name of this other STI/STD *(Use the keyboard to type or click letters on the screen)*  [DISI1]

_____________

- Don’t know

1. Please specify the first time you had it [DISI2]

______ (year) ______ (month)

1. Don’t know

**[Check: should be later than year of birth, as well as, before date of interview 🡪 loop back to question 65I.2]**

**Message:**

The year of first time you had other STI/STD should be later than the year of your birth, as well as, before today. Please correct it.

1. Have you ever had hepatitis B? [DISJ]
2. No
3. Yes **🡪 go to question 65J.1**
4. Don’t know
5. Refuse to answer
6. Please specify the first time you had hepatitis B [DISJ1]

______ (year) ______ (month)

1. Don’t know

**[Check: should be later than year of birth, as well as, before date of interview 🡪 loop back to question 65J.1]**

**Message:**

The year of first time you had hepatitis B should be later than the year of your birth, as well as, before today. Please correct it.

1. Have you ever had hepatitis C? [DISK]
2. No
3. Yes **🡪 go to question 65K.1**
4. Don’t know
5. Refuse to answer
6. Please specify the first time you had hepatitis C [DISK1]

______ (year) ______ (month)

1. Don’t know

**[Check: should be later than year of birth, as well as, before date of interview 🡪 loop back to question 65K.1]**

**Message:**

The year of first time you had hepatitis C should be later than the year of your birth, as well as, before today. Please correct it.

1. Have you ever had hepatitis of an unknown type? [DISL]
2. No
3. Yes **🡪 go to question 65L.1**
4. Don’t know
5. Refuse to answer
6. Please specify the first time you had hepatitis of an unknown type

[DISL1]

______ (year) ______ (month)

1. Don’t know

**[Check: should be later than year of birth, as well as, before date of interview 🡪 loop back to question 65L.1]**

**Message:**

The year of first time you had hepatitis of an unknown type should be later than the year of your birth, as well as, before today. Please correct it.

1. Symptoms
   1. **During the past 6 months**, how often have you had pain or burning in the genital or anal area? [SYMPT1]
2. Never
3. Sometimes
4. Often
5. Always
6. Don’t know
7. Refuse to answer
   1. **During the past 6 months**, how often have you had itching in the genital or anal area? [SYMPT2]
8. Never
9. Sometimes
10. Often
11. Always
12. Don’t know
13. Refuse to answer
    1. **During the past 6 months**, how often have you had sores, pimples or other lesions in the genital or anal area? [SYMPT3]
14. Never
15. Sometimes
16. Often
17. Always
18. Don’t know
19. Refuse to answer
    1. **During the past 6 months**, how often have you had warts in the genital or anal area? [SYMPT4]
20. Never
21. Sometimes
22. Often
23. Always
24. Don’t know
25. Refuse to answer
    1. **During the past 6 months**, how often have you had discharge from the opening of your penis? [SYMPT5]
26. Never
27. Sometimes
28. Often
29. Always
30. Don’t know
31. Refuse to answer
    1. **During the past 6 months**, how often have you had pain when urinating (peeing)? [SYMPT6]
32. Never
33. Sometimes
34. Often
35. Always
36. Don’t know
37. Refuse to answer
    1. **During the past 6 months**, how often have you had blood, pus or discoloration in your urine? [SYMPT7]
38. Never
39. Sometimes
40. Often
41. Always
42. Don’t know
43. Refuse to answer
    1. **During the past 6 months**, how often has it been hard to urinate? [SYMPT8]
44. Never
45. Sometimes
46. Often
47. Always
48. Don’t know
49. Refuse to answer
    1. **During the past 6 months**, how often have you had pain during sex? [SYMPT9]
50. Never
51. Sometimes
52. Often
53. Always
54. Don’t know
55. Refuse to answer

**[If none of above symptoms 🡪 go to question 68]**

1. What did you do about the above-mentioned symptoms? Did you… ***(click all that apply)***

[DLSYMP]

1. Go to a special STI clinic for treatment
2. Go to a general hospital for treatment
3. Go to a private clinic for treatment
4. Bought medicine on my own without seeing a medical professional
5. Other **🡪 go to question 67.1**
6. Did nothing
7. Don’t know
8. Refuse to answer
   1. Please specify where you went for treatment *(Use the keyboard to type or click letters on the screen)* [DLSYMP1]

___________________________

- Don’t know
- Refuse to answer

1. Testing place for STIs
   1. How comfortable would you be receiving testing and counseling for sexually transmitted infections **in a gay bar**? [PLCSTI1]
2. Very comfortable
3. Comfortable
4. Neutral
5. Uncomfortable
6. Very uncomfortable
7. Don’t know
8. Refuse to answer
   1. How comfortable would you be receiving testing and counseling for sexually transmitted infections **in a family doctor’s office**? [PLCSTI2]
9. Very comfortable
10. Comfortable
11. Neutral
12. Uncomfortable
13. Very uncomfortable
14. Don’t know
15. Refuse to answer
    1. How comfortable would you be receiving testing and counseling for sexually transmitted infections **in a sexual health clinic**? [PLCSTI3]
16. Very comfortable
17. Comfortable
18. Neutral
19. Uncomfortable
20. Very uncomfortable
21. Don’t know
22. Refuse to answer
    1. How comfortable would you be receiving testing and counseling for sexually transmitted infections **in a clinic for gay men only**? [PLCSTI4]
23. Very comfortable
24. Comfortable
25. Neutral
26. Uncomfortable
27. Very uncomfortable
28. Don’t know
29. Refuse to answer
    1. How comfortable would you be receiving testing and counseling for sexually transmitted infections **in a bathhouse**? [PLCSTI5]
30. Very comfortable
31. Comfortable
32. Neutral
33. Uncomfortable
34. Very uncomfortable
35. Don’t know
36. Refuse to answer
    1. How comfortable would you be receiving testing and counseling for sexually transmitted infections **in an anonymous testing clinic**? [PLCSTI6]
37. Very comfortable
38. Comfortable
39. Neutral
40. Uncomfortable
41. Very uncomfortable
42. Don’t know
43. Refuse to answer
44. Have you ever been vaccinated against hepatitis B? [VACHPB]
45. No
46. Yes **🡪 go to question 69.1**
47. Don’t know
48. Refuse to answer
    1. How many shots did you get (hepatitis B or combined hepatitis A and hepatitis B)?

[VACHPB1]

1. One shot
2. Two shots
3. Three shots
4. Don’t know

**HIV testing history**

*The following questions ask about HIV testing.* [Info22]

1. Have you ever been tested for HIV? [HIVTST]
2. No **🡪 go to question 78**
3. Yes
4. Don’t know **🡪 go to question 79**
5. Refuse to answer **🡪 go to question 79**

If yes:

1. How many times in all have you been tested for HIV? *If you don’t remember or don’t know exactly, please click the “Don’t know” button.* [NHIVTST]

_____ (times) (range 1 - 99)

1. Don’t know **🡪 go to question 71.1**
2. Refuse to answer **🡪 go to question 71.1**
   1. If you don’t remember exactly, check one of the following that fits to your situation: [NHIVTST1]
3. Less than 5
4. 5 to 9
5. 10 to 19
6. 20 to 29
7. 30 or more
8. Don’t know (Don’t remember) **🡪 go to question 73**
9. Refuse to answer **🡪 go to question 73**

**[If only tested once 🡪 go to question 73]**

1. If more than one test,
   1. When was the first time that you were tested for HIV? [DTFRST]

________ (year) _______ (month) (range 01/01/1980 - current)

1. Don’t know
2. Refuse to answer

- 1. In what country did you have your first HIV test? ***(click only one answer)***

[PLCFRST]

1. Canada
2. Other country **🡪 go to question 72.2a**
3. Don’t know
4. Refuse to answer
   - 1. Please enter the country where you had your first HIV test *(Use the keyboard to type or click letters on the screen)*  [PLCFRST1]

____________________

- Don’t know
- Refuse to answer

1. Details concerning the most recent HIV test
   1. When was the most recent time that you were tested for HIV? [DTTST1]

________ (year) _______ (month) (range 01/01/1980 - current)

1. Don’t know
2. Refuse to answer
   1. In what country/province/city did you have your most recent HIV test? *(Use the keyboard to type or click letters on the screen)* [PLCETST1]

____________________

- Don’t know
- Refuse to answer
  1. Where did you have your most recent test?  ***(click only one answer)*** [SETTST1]

1. Doctor’s office/clinic
2. Walk-in clinic
3. Hospital, inpatient
4. Hospital, outpatient/emergency room
5. Community health center
6. Special clinic for HIV testing
7. Immigration doctor
8. Other **🡪 go to question 73.3a**
9. Don’t know
10. Refuse to answer
    - 1. Please specify any other place you had your most recent HIV test *(Use the keyboard to type or click letters on the screen)* [SETTST1A]

____________________

- Don’t know
- Refuse to answer
  1. What made you decide to have your most recent HIV test? ***(click all of the reasons that apply)*** [RSHIV]

1. You think you might have been exposed to HIV through sexual activity
2. You think you might have been exposed to HIV through sharing needles
3. You think you might have been exposed to HIV through a blood transfusion
4. Your sexual partner was/is HIV-positive
5. You wanted to make sure you were HIV negative so you and your partner could have sex without condoms
6. You were starting a new sexual relationship
7. You had a “flu-like” illness and thought it might be HIV infection
8. You always get tested (routinely)
9. You just wanted to know/You were curious
10. The doctor offered it to you
11. For immigration purposes
12. Your insurance company suggested it
13. Other **🡪 go to question 73.4a**
14. Don’t know
    - 1. Refuse to answer
      2. Please specify other reason for your most recent HIV test *(Use the keyboard to type or click letters on the screen)* [RSHIV1]

________________________________________

- Don’t know
- Refuse to answer
  1. What was the result of your most recent HIV test? [RSLTTST1]

1. HIV-negative **🡪 go to question 73.5.2**
2. HIV-positive
3. Don’t know **🡪 go to question 74**
4. Refuse to answer **🡪 go to question 74**

73.5.1 If HIV-positive in the most recent test,

73.5.1a What was the date of your first HIV-positive test? [DTFRSTP]

________ (year) _______ (month)

1. Don’t know
2. Refuse to answer

73.5.1b Had you ever had a HIV-negative test before your first HIV-positive test?

[NEGBFPOS]

1. No **🡪 go to question 73.5c**
2. Yes
3. Don’t know **🡪 go to question 73.5c**
4. Refuse to answer **🡪 go to question 73.5c**

If yes:

73.5.1b1 What was the date of your last HIV-negative test? [DTLASTN]

________ (year) _______ (month)

1. Don’t know
2. Refuse to answer

73.5.1c In what year, do you think you became infected with HIV? [INFECT1]

________ (year)

1. Don’t know
2. Refuse to answer

73.5.1d How do you think you became infected? ***(click all that apply)*** [INFECT2]

1. Sex with a man **🡪 go to question 73.5.1d2**
2. Sex with a woman **🡪 go to question 73.5.1d2**
3. Injecting drugs  **🡪 go to question 73.5.1e**
4. Blood transfusion **🡪 go to question 73.5.1e**
5. Needlestick injury **🡪 go to question 73.5.1e**
6. Other **🡪 go to question 73.5.1d1**
7. Don’t know **🡪 go to question 73.5.1e**
8. Refuse to answer **🡪 go to question 73.5.1e**

73.5.1d1 Please specify other sources of your HIV infection *(Use the keyboard to type or click letters on the screen)* [INFECT3]

_____________________________

- - - - - Don’t know
        - Refuse to answer

73.5.1d2 You indicated you think you were infected through sex with a man or a woman; what was your relationship with this partner?

[INFECT4]

1. Regular male partner
2. Regular female partner
3. Casual male partner
4. Casual female partner
5. Client
6. Sex worker
7. Don’t know
8. Refuse to answer

73.5.1d3 What makes you think this partner may have infected you? ***(click all that apply)***  [INFECT5]

1. This partner was HIV-infected
2. This partner had injected drugs
3. This partner was from Africa or the Caribbean
4. This partner had had sex with another man
5. This partner had sex with a woman with known HIV infection
6. This partner had sex with a man with known HIV infection
7. This partner was in a relationship with a partner at high risk for HIV infection
8. This partner had sex with a sex trade worker
9. This partner had had a blood transfusion
10. This partner had received clotting factors (hemophilia)
11. Other **🡪 go to question 73.5d4**
12. Don’t know
13. Refuse to answer

73.5.1d4 Please specify other reasons that you think this partner may have infected you *(Use the keyboard to type or click letters on the screen)* [INFECT6]

_____________________________

- Don’t know
- Refuse to answer

73.5.1e After you tested HIV-positive, did you tell your regular male sexual partners about your HIV status? [POSTLDRM]

1. None of them
2. Some of them
3. All of them
4. Don’t know
5. Refuse to answer
6. Not applicable

73.5.1f After you tested HIV-positive, did you tell your regular female sexual partners about your HIV status? [POSTLDRW]

1. None of them
2. Some of them
3. All of them
4. Don’t know
5. Refuse to answer
6. Not applicable

73.5.1g After you tested HIV-positive, did you tell your casual male sexual partners about your HIV status? [POSTLDCM]

1. None of them
2. Some of them
3. All of them
4. Don’t know
5. Refuse to answer
6. Not applicable

73.5.1h After you tested HIV-positive, did you tell your casual female sexual partners about your HIV status? [POSTLDCW]

1. None of them
2. Some of them
3. All of them
4. Don’t know
5. Refuse to answer
6. Not applicable

73.5.1i How much more likely are you to have anal sex without a condom with a casual partner who is HIV-positive? [POSPTPOS]

1. Much more likely
2. More likely
3. About as likely
4. Less likely
5. Much less likely
6. Don’t know
7. Refuse to answer
8. Not applicable

**🡪 go to question 74**

73.5.2 If HIV-negative in the most recent test,

73.5.2a *Recently, a study among HIV-uninfected persons given anti-HIV drugs on a regular basis reported some protection (40-70%) from becoming infected with HIV; this is called Pre-Exposure Prophylaxis (PrEP).* Thinking about your risk of HIV, how likely would you be to take PrEP?

[NEGPREP]

1. Highly likely
2. Very likely
3. Somewhat likely
4. Somewhat unlikely **🡪 go to question 73.5.2a1**
5. Very unlikely **🡪 go to question 73.5.2a1**
6. Highly unlikely **🡪 go to question 73.5.2a1**
7. Don’t know
8. Refuse to answer

73.5.2a1 Why are you unlikely to take PrEP? ***(click all that apply)***

[NGPREP1]

1. My risk of HIV is too low
2. PrEP probably doesn’t work
3. It would be a burden to have to take pills every day
4. I would be concerned about possible side-effects
5. Other **🡪 go to question 73.5.2a2**
6. Don’t know
7. Refuse to answer

73.5.2a2 Please indicate why you are unlikely to take PrEP *(Use the keyboard to type or click letters on the screen)*

[NGPREP2]

_____________________________

- Don’t know
- Refuse to answer

73.5.2b *In Ontario, some HIV-positive persons have been prosecuted (i.e. put on trial) for having sex without disclosing their HIV status.* How much would a concern about being prosecuted affect your decision to get tested for HIV? [NEGCRIM]

1. Much less likely to get tested
2. Less likely to get tested
3. No change in how likely I am to get tested
4. More likely to get tested
5. Much more likely to get tested
6. Don’t know
7. Refuse to answer

73.5.2c In the past 6 months when you had sex with a casual partner, how often did you ask your partner about their HIV status before having anal sex?

[NEGPTSTA]

1. Never
2. Rarely
3. Some of the time
4. Most of the time
5. All the time
6. Don’t know
7. Refuse to answer
8. Not applicable

73.5.2d How much more likely are you to have anal sex without a condom with a casual partner who is HIV-negative? [NEGPTNEG]

1. Much more likely
2. More likely
3. About as likely
4. Less likely
5. Much less likely
6. Don’t know
7. Refuse to answer
8. Not applicable

73.5.2e How likely do you think it is that you will become infected with HIV?

[NGINFECT]

1. Certain or almost certain
2. Highly likely
3. Somewhat likely
4. Somewhat unlikely
5. Highly unlikely
6. Not at all
7. Don’t know
8. Refuse to answer

**If had casual partner during the past 6 months (CASPTN =1)**

73.5.2f In the past 6 months, have you used condoms during anal sex with all of your casual partners? [NEGCND]

- 1. No
  2. Yes **🡪 go to question 74**

1. Don’t know **🡪 go to question 74**
2. Refuse to answer **🡪 go to question 74**

73.5.2f1 You don’t use condoms all the time because… ***(click all that apply)***  [NEGCND1]

1. You feel you are at low risk for getting HIV

**🡪 go to question 73.5.2f2**

1. You are not concerned with getting HIV

**🡪 go to question 73.5.2f4**

1. You are concerned with getting HIV but it is hard to remember to use a condom in the heat of the moment
2. You get a certain pleasure in doing something you are not supposed to do
3. You are tired of using condoms
4. Pleasure without a condom is more important to you
5. Don’t know
6. Refuse to answer

73.5.2f2 You indicated that you don’t use condoms all the time because you feel you are at low risk for getting HIV because… ***(click all that apply)***  [NEGCND2]

1. Most people in your area don’t have HIV
2. None of your partners are HIV-positive
3. HIV is difficult to transmit
4. After all you have done you haven’t gotten HIV, so maybe you are immune
5. Other **🡪 go to question 73.5.2f3**
6. Don’t know
7. Refuse to answer

73.5.2f3 Please indicate why you feel you are at low risk for getting HIV *(Use the keyboard to type or click letters on the screen)* [NEGCND3]

_____________________________

- Don’t know
- Refuse to answer

73.5.2f4 You indicated that you don’t use condoms all the time because you are not concerned with getting HIV because… ***(click all that apply)***  [NEGCND4]

1. Your chances of getting HIV are so high that practicing safe sex doesn’t make much difference
2. There isn’t much to look forward to when you get old, so you might as well have fun now
3. You don’t care so much if you get infected because effective medications are available
4. Don’t know
5. Refuse to answer

**[If born in Canada 🡪 go to question 75]**

1. How many times have you been tested for HIV for immigration purposes? [NIMMTST]

**______** (range 0 – 99)

1. Don’t know **🡪 go to question 75**
2. Refuse to answer **🡪 go to question 75**

**[If number of tests = 0 🡪 go to question 75]**

- 1. Details of your most recent test for immigration purpose
     1. When was the most recent time you were tested for HIV for immigration purposes? [DTIMM1]

________ (year) _______ (month) (range 01/01/1980 - current)

1. Don’t know
2. Refuse to answer
   - 1. In what country/city did you have your most recent HIV test for immigration purposes? *(Use the keyboard to type or click letters on the screen)* [PLCEIMM1]

____________________

- Don’t know
- Refuse to answer
  - 1. What was the result of your most recent HIV test for immigration purposes? [RSLTIMM1]

1. HIV-negative
2. HIV-positive
3. Don’t know
4. Refuse to answer
5. Have you ever taken special medications to reduce the risk of HIV infection following possible exposure? *(e.g. post-exposure treatment)*  [ARTPRVT]
6. No
7. Yes, but not in the past 6 months
8. Yes, in the past 6 months
9. Don’t know
10. Refuse to answer

**[If the most recent test is negative 🡪 go to question 77]**

1. Have you ever taken HIV medications to treat HIV infection? [ARTTXT]
2. No **🡪 go to question 76.1**
3. Yes, but not in the past 6 months **🡪 go to question 77**
4. Yes, in the past 6 months **🡪 go to question 77**
5. Don’t know **🡪 go to question 77**
6. Refuse to answer **🡪 go to question 77**
   1. Do you visit a doctor regularly to monitor your HIV infection (e.g. to follow your viral load and CD4 count)? [ACCESS]
7. No **🡪 go to question 76.1a**
8. Yes **🡪 go to question 77**
9. Don’t know **🡪 go to question 77**
10. Refuse to answer **🡪 go to question 77**

If no,

- - 1. Why don’t you see a doctor to monitor your HIV infection on a regular basis? ***(click all that apply)***  [ACCESS1]

1. My regular doctor has no experience with this type of care
2. I don’t know where to go
3. I can’t afford the expense (e.g for transport, child care)
4. I have no time
5. I feel fine and don’t think I need to monitor my HIV infection
6. I don’t believe HIV drugs are safe and effective
7. I am worried someone might find out I am HIV-positive
8. Other **🡪 go to question 76.1b**
9. Don’t know
10. Refuse to answer
    - 1. Please specify any other reason that you don’t see a doctor to monitor your HIV infection on a regular basis? *(Use the keyboard to type or click letters on the screen)* [ACCESS2]

____________________

- Don’t know
- Refuse to answer

**[If both no or Don’t know or refuse in question 75 and question 76**

**🡪 go to question 79]**

1. If yes for question 75 or question 76,
   1. How did you pay for your HIV medications? ***(click all that apply)*** [PAYART]
2. Trillium Drug Program (TDP)
3. Ontario Drug Benefit Program (ODB)
4. Compassionate release program
5. PWA program
6. Expanded access protocol
7. Research study
8. Private insurance
9. Out-of-pocket
10. Other **🡪 go to question 77.1a**
11. Don’t know
12. Refuse to answer
    - 1. Please specify other sources for HIV medication *(Use the keyboard to type or click letters on the screen)*  [PAYART1]

______________________________

- Don’t know
- Refuse to answer
  1. When did you first start taking HIV medications? *(Please make your best guess if you Don’t know for sure.)* [FRSTART]

________ (year) _______ (month) (range 01/01/1980 - current)

1. Don’t know
2. Refuse to answer
   1. Are you now taking HIV medications? [ARTNOW]
3. No
4. Yes **🡪 go to question 79**
5. Don’t know **🡪 go to question 79**
6. Refuse to answer **🡪 go to question 79**

If no,

- 1. When did you last take HIV medications? [LASTART]

________ (year) _______ (month) (range 01/01/1980 - current)

1. Don’t know
2. Refuse to answer

**🡪 go to question 79**

1. You have never been tested for HIV; why were you not tested? ***(next screen has additional reasons) (click all reasons that apply)*** [NOHIV1]
2. I have not done anything that makes me think I may have HIV
3. If I tested positive, nothing can be done
4. I do not want to know
5. I am afraid to know whether I am HIV positive
6. If I tested positive, I could be criminally prosecuted
7. I don’t think I can get HIV
8. I think I am HIV-positive
9. I think I am HIV-negative
10. I always have “safe’” sex
11. I never thought about getting a test
12. I am worried about the impact of being HIV-positive on my sex life
13. I don’t think the test is always accurate
14. I have never had sex with an infected person
15. I am healthy so I don’t need to be tested
16. Don’t know
17. Refuse to answer **🡪 go to question 79**
    1. Why were you not tested? ***(continued) (click all reasons that apply)*** [NOHIV2]
18. I could not deal with knowing I was infected
19. I do not know where to get tested
20. I am afraid of having my name reported
21. I do not have health care coverage in Canada
22. I am worried about being discriminated against
23. Testing positive could affect my career or insurance
24. Testing positive could affect my relationships
25. I am afraid of needles
26. I want to be tested, I just haven’t done it yet
27. Doesn’t matter if I’m infected because of my age
28. I do not believe that HIV causes AIDS
29. I do not know anyone who has HIV or AIDS so I am not worried
30. I don’t have a doctor
31. I don’t have time to see a doctor
32. Other
    1. Please specify other reasons for not having an HIV test *(Use the keyboard to type or click letters on the screen)* [NOHIVOTH]

___________________________

- Don’t know
- Refuse to answer

**SECTION 5: OTHER HEALTH BEHAVIOURS**

*The following section asks about your other health behaviours and use of health care services.*

**Health behaviours**

*First, we will ask about your other health behaviours.* [Info23]

1. Have you ever smoked? [SMOKE]
2. Never **🡪 go to question 80**
3. Yes, but I do not smoke any more
4. Yes and I smoke now
5. Don’t know
6. Refuse to answer **🡪 go to question 80**

If ever smoked,

- 1. At what age did you start smoking? [SMOKE1]

______ (age in years) (range 0 – 80)

1. Don’t know
2. Refuse to answer
3. Refuse to answer

**[Check: should be younger than or equal to current age 🡪 loop back to question 79.1]**

**Message:**

Your age when you started smoking should be younger than your current age. Please correct it.

- 1. At the present time, how often do you smoke cigarettes? [SMOKE2]

1. Not at all **🡪 go to question 79.2b**
2. Occasionally **🡪 go to question 80**
3. Every day **🡪 go to question 79.2a**
4. Don’t know **🡪 go to question 80**
5. Refuse to answer **🡪 go to question 80**

If every day,

- - 1. Please specify the usual number of cigarettes you smoke each day: [SMOKE2A]

_____________ (range 1 – 99)

1. Don’t know
2. Refuse to answer

**🡪 go to question 80**

If not at all

- - 1. At what age did you stop? [SMOKE2B]

______ (age in years) (range 0 –80)

1. Don’t know
2. Refuse to answer

**[Check: should be younger than or equal to current age 🡪 loop back to question 79.2b]**

**Message:**

Your age when you stopped smoking should be younger than your current age. Please correct it.

1. **During the past 6 months**, on average how often did you drink alcohol? *(By alcohol we mean beer, wine or liquor. By a drink, we mean: one bottle, can or a glass of beer; one glass of wine or wine cooler; one mixed drink or cocktail)* [DRINK]
2. Never **🡪 go to question 83**
3. Less than once a month
4. Once a month
5. 2 to 3 times a month
6. Once a week
7. 2 to 3 times a week
8. 4 to 6 times a week
9. Daily
10. Don’t know
    - 1. Refuse to answer

If daily,

- 1. How many drinks per day do you typically have? [DRINK1]

______ (range: 1 - 99)

1. Don’t know
2. Refuse to answer
3. **During the past 6 months,** have you ever had 5 or more drinks of alcohol within a few hours? [DRINK2]
4. Never
5. Less than once a month
6. Once a month
7. 2 to 3 times a month
8. Once a week
9. 2 to 3 times a week
10. 4 to 6 times a week
11. Daily
12. Don’t know
    - 1. Refuse to answer

1. **During the past 6 months,** have you ever had sex while you were drinking such that it may have affected your decision to use a condom? [DRINK3]
2. Never
3. Occasionally
4. Sometimes
5. Often
6. Most of the time
7. Don’t know
8. Refuse to answer
9. Have you ever used any of the following recreational/illicit drugs? ***(click all that apply)***

[DRUG]

1. Marijuana, cannabis or hashish (‘pot’, ‘weed’, ‘dope’, ‘grass’)
2. Cocaine (‘crack’, ‘coke’, ‘freebase’)
3. Methamphetamines or crystal meth (‘speed’, ‘chalk’, ‘ice’, ‘glass’)
4. Heroin or opiates (‘smack’, ‘junk’)
5. Amyl nitrite or Poppers (‘rush’, ‘snappers’)
6. Ecstasy or MDMA (‘E’, ‘XTC’)
7. Psychedelics, hallucinogens, PCP, LSD or mescaline (‘mushrooms’, ‘acid’)
8. Ketamine (‘special K’, ‘K’, ‘ket’, ‘vitamin K’)
9. Sniff glue, gasoline, or other solvents
10. Khat (‘chat’, ‘qat’, ‘ghat’, ‘gaat’, ‘jaad’, ‘miraa’)
11. GHB (‘G’, ‘liquid E’, ‘liquid X’)
12. Other **🡪 go to question 83.1**
13. None of the above **🡪 go to question 85**
14. Don’t know
    1. Refuse to answer
    2. Please specify other drugs you have used *(Use the keyboard to type or click letters on the screen)* [DRUG1]

_________________

- Don’t know
- Refuse to answer

1. **During the past 6 months,** have you ever used any of the following recreational/illicit drugs? ***(click all that apply)***  [DRUG6M]
2. Marijuana, cannabis or hashish (‘pot’, ‘weed’, ‘dope’, ‘grass’)
3. Cocaine (‘crack’, ‘coke’, ‘freebase’)
4. Methamphetamines or crystal meth (‘speed’, ‘chalk’, ‘ice’, ‘glass’)
5. Heroin or opiates (‘smack’, ‘junk’)
6. Amyl nitrite or Poppers (‘rush’, ‘snappers’)
7. Ecstasy or MDMA (‘E’, ‘XTC’)
8. Psychedelics, hallucinogens, PCP, LSD or mescaline (‘mushrooms’, ‘acid’)
9. Ketamine (‘special K’, ‘K’, ‘ket’, ‘vitamin K’)
10. Sniff glue, gasoline, or other solvents
11. Khat (‘chat’, ‘qat’, ‘ghat’, ‘gaat’, ‘jaad’, ‘miraa’)
12. GHB (‘G’, ‘liquid E’, ‘liquid X’)
13. Other **🡪 go to question 84.1**
14. None of the above
15. Don’t know
16. Refuse to answer
    1. Please specify other drugs you have used **during the past 6 months** *(Use the keyboard to type or click letters on the screen)* [DRUG6M1]

_________________

- Don’t know
- Refuse to answer

1. Have you ever injected any of the following recreational/illicit drugs? ***(click all that apply)***  [INJDRG]
2. Heroin
3. Cocaine
4. Crystal meth
5. Other **🡪 go to question 85.1**
6. None of the above **🡪 go to question 87**
7. Don’t know
8. Refuse to answer
   1. Please specify other drugs you have ever injected *(Use the keyboard to type or click letters on the screen)* [INJDRG1]

_________________

- Don’t know
- Refuse to answer

1. **During the past 6 months,** have you ever injected any of the following recreational/illicit drugs? ***(click all that apply)***  [INJD6M]
2. Heroin
3. Cocaine
4. Crystal meth
5. Other **🡪 go to question 86.1**
6. None of the above
7. Don’t know
8. Refuse to answer
   1. Please specify other drugs you have injected **during the past 6 months** *(Use the keyboard to type or click letters on the screen)* [INJD6M1]

_________________

- Don’t know
- Refuse to answer

1. **During the past 6 months,** how often did you use any of the previously mentioned drugs within 2 hours before sex or during sex (oral, vaginal or anal)? [DRUGSEX]
2. Never
3. Occasionally
4. Sometimes
5. Often
6. Most of the time
7. Don’t know
8. Refuse to answer
9. Have you ever used a needle or other shooting equipment (such as cookers, cotton or water) that had already been used by someone else? [SHAREEQP]
10. Never
11. Occasionally
12. Sometimes
13. Often
14. Most of the time
15. Don’t know
16. Refuse to answer

**Health status and health care access**

*The following questions ask about your health status and use of health care services.* [Info24]

1. What type of health care insurance do you have? ***(click only one answer, i.e. indicate the first one that applies)*** [INSURA]
2. Ontario Health Insurance Plan (OHIP)
3. Interim Federal Health (IFH)
4. Private insurance
5. None
6. Don’t know
7. Refuse to answer
8. In general, compared to others of your age, would you say your health is … ***(click the best answer)***  [HEALTH]
9. Excellent
10. Very good
11. Good
12. Fair
13. Poor
14. Don’t know
15. Refuse to answer
16. Do you have a family doctor? *(i.e. a* *general or family practitioner who provides you with your usual medical care)*  [FMLYDOC]
17. No
18. Yes
19. Don’t know
20. Refuse to answer
21. **During the past 6 months**, have you seen any of the following health or social service professionals? ***(click all that apply)*** [HLTHSEV]
22. Walk-in clinic
23. Emergency room
24. Family doctor or GP
25. Eye specialist or ophthalmologist
26. Other specialist (such as surgeon, allergist, orthopedist, gynecologist, etc)
27. HIV specialist
28. Psychiatrist
29. Nurse or nurse practitioner
30. Dentist
31. Chiropractor
32. Physiotherapist
33. Psychologist
34. Social worker or counselor
35. Dietician or nutritionist
36. Speech therapist, audiologist, or occupational therapist
37. Other **🡪 go to question 92.1**
38. None of the above
39. Don’t know
40. Refuse to answer
    1. Please specify other health or social services you used during the past 6 months *(Use the keyboard to type or click letters on the screen)* [HLTHSEV1]

___________________

- Don’t know
- Refuse to answer

1. **During the past 6 months**, have you consulted any of the following alternative health care professionals about your physical or mental health? ***(click all that apply)***

[OTHSEV]

1. Massage therapist
2. Acupuncturist
3. Homeopath, naturopath or herbalist
4. Religious or spiritual healer
5. Traditional healer
6. Other **🡪 go to question 93.1**
7. None of the above
8. Don’t know
9. Refuse to answer
   1. Please specify other alternative health care professionals you used during the past 6 months *(Use the keyboard to type or click letters on the screen)* [OTHSEV1]

___________________

- Don’t know
- Refuse to answer

1. Has a health professional, such as a doctor, nurse, or other health care specialist **ever** told you that you have one of the following conditions? ***(click all that apply)*** [OTHDIS]
2. High blood pressure
3. Tuberculosis
4. Asthma / chronic bronchitis
5. Arthritis / rheumatism
6. Back problems
7. Diabetes
8. Heart disease
9. Stomach or digestive problems
10. Depression
11. Any other long-term (chronic) condition that has been diagnosed by a health professional? **🡪 go to question 94.1**
12. None of the above
13. Don’t know
14. Refuse to answer
    1. Please specify other long-term (chronic) conditions *(Use the keyboard to type or click letters on the screen)* [OTHDIS1]

___________________

- Don’t know
- Refuse to answer

1. Have you ever had a surgical operation? [SURGRY]
2. No **🡪 go to question 96**
3. Yes
4. Don’t know **🡪 go to question 96**
5. Refuse to answer **🡪 go to question 96**

If yes,

- 1. How many surgical operations have you had? [NSURGRY]

___________________ (range 1 – 99)

1. Don’t know
2. Refuse to answer
   1. Details of your most recent surgical operation
      1. Please indicate the procedure (type of operation) of your most recent operation *(Use the keyboard to type or click letters on the screen)*

[PRCSURG]

________________

- Don’t know
- Refuse to answer
  - 1. Please enter the year of your most recent surgical operation [DTSURG]

________ (year)

1. Don’t know
2. Refuse to answer

**[Check: should be later than year of birth, as well as, before date of interview 🡪 loop back to question 95.2b]**

**Message:**

The year of your most recent surgical operation should be later than the year of your birth, as well as, before today. Please correct it.

- - 1. In which country did you have your most recent surgical operation? ***(click only one answer)*** [CTRYSUR]

1. Canada
2. Other country **🡪 go to question 95.2c1**
3. Don’t know
4. Refuse to answer
   - - 1. Please enter the country where you had your most recent surgical operation *(Use the keyboard to type or click letters on the screen)*  [CTRYSUR1]

____________________

- Don’t know
- Refuse to answer

1. Have you ever received a blood transfusion (including red cells, plasma, etc)? [TRANS]
2. No **🡪 go to question 97**
3. Yes
4. Don’t know **🡪 go to question 97**
5. Refuse to answer **🡪 go to question 97**

If yes,

- 1. How many times have you received a blood transfusion (including red cells, plasma, etc)? [NTRANS]

**______** (range 1 – 9999)

1. Don’t know
2. Refuse to answer
   1. Details of you most recent transfusion
      1. What kind of blood did you receive in your most recent transfusion?

[TPTRANSL]

1. Your own blood
2. Donor blood
3. Don’t know
4. Refuse to answer
   - 1. Please enter the year of your most recent transfusion [DTTRANSL]

________ (year)

1. Don’t know
2. Refuse to answer

**[Check: should be later than year of birth, as well as, before date of interview 🡪 loop back to question 96.2b]**

**Message:**

The year of your most recent transfusion should be later than the year of your birth, as well as, before today. Please correct it.

- - 1. In which country did you receive your most recent transfusion? ***(click only one answer)*** [CTRANSL]

1. Canada
2. Other country **🡪 go to question 96.2c1**
3. Don’t know
4. Refuse to answer
   - - 1. Please enter the country where you received your most recent transfusion *(Use the keyboard to type or click letters on the screen)* [CTRANSL1]

____________________

- Don’t know
- Refuse to answer

**[If number of tests = 1 or number of tests = REF 🡪 go to question 97]**

- 1. Details of your first transfusion
     1. What kind of blood did you receive in your first transfusion? [TPTRANSF]

1. Your own blood
2. Donor blood
3. Don’t know
4. Refuse to answer
   - 1. Please enter the year of your first transfusion [DTTRANSF]

________ (year)

1. Don’t know
2. Refuse to answer

**[Check: should be later than year of birth, as well as, before date of interview 🡪 loop back to question 96.3b]**

**Message:**

The year of your first transfusion should be later than the year of your birth, as well as, before today. Please correct it.

- - 1. In which country did you receive your first transfusion? ***(click only one answer)*** [CTRANSF]

1. Canada
2. Other country **🡪 go to question 96.3c1**
3. Don’t know
4. Refuse to answer
   - - 1. Please enter the country where you received your first transfusion *(Use the keyboard to type or click letters on the screen)* [CTRANSF1]

____________________

- Don’t know
- Refuse to answer

1. Have you been circumcised? [CIRCUM]
2. No **🡪 go to question 98**
3. Yes
4. Don’t know
5. Refuse to answer **🡪 go to question 98**

If yes,

- 1. In what country were you circumcised? *(Use the keyboard to type or click letters on the screen)* [CIRCUM1]

________________

- Don’t Know
- Refuse to Answer

- 1. What was the reason for your circumcision? ***(click one best answer)*** [CIRCUM2]

1. Medical reason
2. To prevent HIV or other infection
3. Religious reasons
4. Cultural but non-religious reason (e.g. rite of passage)
5. Other **🡪 go to question 97.2a**
6. Don’t know
7. Refuse to answer
   - 1. Please specify other reasons why you had a circumcision *(Use the keyboard to type or click letters on the screen)* [CIRCUM2A]

________________

- Don’t know
- Refuse to Answer
  1. What was your approximate age when you were circumcised? [CIRCUM3]

1. Less than 1 year of age
2. 1 to 12 years of age
3. 13 to 20 years of age
4. 21 years of age or older
5. Don’t know
6. Refuse to answer
7. Have you ever had a tattoo? [TATTOO]
8. No **🡪 go to question 99**
9. Yes
10. Don’t know
11. Refuse to answer **🡪 go to question 99**

If yes,

- 1. How many tattoos have you had? [TATTOO1]

_______ (range 1 – 9999)

1. Don’t know
2. Refuse to answer
   1. In which country or countries were you tattooed? ***(click all that apply)***

[TATTOO2]

1. Canada
2. Other country
3. Don’t know
4. Refuse to answer

If in Canada,

- 1. Please enter the year that you were tattooed in Canada; if more than one, indicate the years separated by a comma “,” *(Use the keyboard to type or click letters on the screen)* [TATTOO3]

___________________

- Don’t know
- Refuse to answer
  1. If in another country or countries,
     1. Please enter these countries, separated by a comma “,” *(Use the keyboard to type or click letters on the screen)* [TATTOO4A]

___________________

- Don’t know
- Refuse to answer
  - 1. Please enter the year that you were tattooed; if more than one, indicate the years separated by a comma “,” *(Use the keyboard to type or click letters on the screen)*  [TATTOO4B]

___________________

- Don’t know
- Refuse to answer

1. Have you had a body piercing? [PIERC]
2. No **🡪 go to question 100**
3. Yes
4. Don’t know
5. Refuse to answer **🡪 go to question 100**

If yes,

- 1. How many body piercings have you had? [PIERC1]

_______ (range 1 – 9999)

1. Don’t know
2. Refuse to answer
   1. In which country or countries did you have a body piercing? ***(click all that apply)***

[PIERC2]

1. Canada
2. Other country
3. Don’t know
4. Refuse to answer

If in Canada,

- 1. Please enter the year(s) that you had body piercings in Canada, separated by a comma “,” *(Use the keyboard to type or click letters on the screen)* [PIERC3]

___________________

- Don’t know
- Refuse to answer
  1. If in another country or countries,
     1. Please enter these countries, separated by a comma “,” *(Use the keyboard to type or click letters on the screen)* [PIERC4A]

___________________

- Don’t know
- Refuse to answer
  - 1. Please enter the year that you had body piercing; if more than one, indicate the years separated by a comma “,” *(Use the keyboard to type or click letters on the screen)* [PIERC4B]

___________________

- Don’t know
- Refuse to answer

1. Have you had scarification? *(i.e. scars made on your face or other body parts, usually related to culture or tribal practices, often in a traditional ceremony)* [SCARIF]
2. No **🡪 go to question 101**
3. Yes
4. Don’t know
5. Refuse to answer **🡪 go to question 101**

If yes,

- 1. In which country or countries was this done? ***(click all that apply)*** [SCARIF1]

1. Canada
2. Other country
3. Don’t know
4. Refuse to answer
   1. If in Canada, please enter the year; if more than one, indicate the years separated by a comma “,” *(Use the keyboard to type or click letters on the screen)* [SCARIF2]

___________________

- Don’t know
- Refuse to answer
  1. If in another country or countries,
     1. Please enter these countries, separated by a comma “,” *(Use the keyboard to type or click letters on the screen)* [SCARIF3A]

___________________

- Don’t know
- Refuse to answer
  - 1. Please enter the year; if more than one, indicate the years separated by a comma “,” *(Use the keyboard to type or click letters on the screen)*

[SCARIF3B]

___________________

- Don’t know
- Refuse to answer

**SECTION 6: HIV and STI KNOWLEDGE, BELIEFS AND ATTITUDES**

*This section will ask you about your knowledge, beliefs and attitudes toward HIV and sexually transmitted infections (STIs).* [Info25]

1. Are you infected with HIV (with or without AIDS)? [HIVINFCT]
2. No
3. Yes **🡪 go to question 101.3**
4. Don’t know
5. Refuse to answer
   1. What do you think the chances are that you will ever get HIV/AIDS? Would you say that it is . . . [CHNCEHIV]
6. Impossible
7. Not likely
8. Somewhat likely
9. Very likely
10. Don’t know
11. Refuse to answer
    1. How concerned are you about becoming infected with HIV? [WORRYHIV]
12. Very worried
13. Somewhat worried
14. Not very worried
15. Not worried at all
16. Don’t know
17. Refuse to answer

**🡪 go to question 102**

- 1. Indicate your level of agreement with the following statement “I am more likely to disclose my HIV-positive status because I am afraid of being prosecuted” :

[POSCRIM1]

- 1. Strongly agree
  2. Agree
  3. Neutral (neither agree nor disagree)
  4. Disagree
  5. Strongly disagree

1. Don’t know
2. Refuse to answer
   1. Indicate your level of agreement with the following statement “I am more likely to use a condom because I am afraid of being prosecuted” : [POSCRIM2]
3. Strongly agree
4. Agree
5. Neutral (neither agree nor disagree)
6. Disagree
7. Strongly disagree
8. Don’t know
9. Refuse to answer
10. What do you think the chances are that you will ever get another sexually transmitted infection (other than HIV)? Would you say that it is . . . [CHNCESTI]
11. Impossible
12. Not likely
13. Somewhat likely
14. Very likely
15. Don’t know
16. Refuse to answer
17. Do you know anyone in Canada who is HIV-infected or has AIDS? *(Include people who have died; do not include yourself if you are infected)*  [KNOWCA]
18. No **🡪 go to question 104**
19. Yes
20. Don’t know **🡪 go to question 104**
21. Refuse to answer **🡪 go to question 104**

If yes,

- 1. Approximately how many people in Canada do you know who have or have had HIV/AIDS? [KNEWCA1]

________  (range 1 – 9999) (in the program, not enforce the range)

1. Don’t know
2. Refuse to answer
   1. How are they related to you?  *(****click all that apply)*** [KNEWCA2]
3. Child
4. Spouse or common-law partner
5. Close family member (parent/brother/sister)
6. Extended family (aunts/uncles/cousins/grandparents/grandchildren)
7. Friend
8. Community member (not a friend)
9. Other **🡪 go to question 103.2a**
10. Don’t know
11. Refuse to answer
    - 1. Please specify other relationships with those infected with HIV *(Use the keyboard to type or click letters on the screen)*  [KNEWCA3]

________________

- Don’t know
- Refuse to answer

**[If born in Canada 🡪 go to question 105]**

1. Do you know anyone in your home country who is HIV-infected or has AIDS? *(Include people who have died)*  [KNOWHM]
   1. No **🡪 go to question 105**
   2. Yes
2. Don’t know **🡪 go to question 105**
3. Refuse to answer **🡪 go to question 105**

If yes,

- 1. Approximately how many people in your home country do you know who have or have had HIV/AIDS? [KNEWHM1]

________ (range 1 – 9999) (in the program, not enforce the range)

1. Don’t know
2. Refuse to answer
   1. How are they related to you? *(****click all that apply)*** [KNEWHM2]
3. Child
4. Spouse or common-law partner
5. Close family member (parents/brothers/sisters)
6. Extended family (aunts/uncles/cousins/grandparents/grandchildren)
7. Friend
8. Community member (not a friend)
9. Other **🡪 go to question 104.2a**
10. Don’t know
11. Refuse to answer
    - 1. Please specify other relationships with those infected with HIV in your home country *(Use the keyboard to type or click letters on the screen)*

[KNEWHM3]

________________

- Don’t know
- Refuse to answer

1. Please indicate whether you agree or disagree with the following statements concerning persons living with HIV/AIDS: [Info26]
2. If a member of your family became infected with HIV, you would want it to remain a secret. [STIGMAA]
3. Strongly agree
4. Agree
5. Neutral (neither agree nor disagree)
6. Disagree
7. Strongly disagree
8. Don’t know
9. Refuse to answer

1. If a teacher has HIV but is not sick, he or she should not be allowed to continue teaching in the school. [STIGMAB]
   1. Strongly agree
   2. Agree
   3. Neutral (neither agree nor disagree)
   4. Disagree
   5. Strongly disagree
2. Don’t know
3. Refuse to answer

1. If a member of your family became sick with HIV, you would be willing to care for him or her in your household. [STIGMAC]
2. Strongly agree
3. Agree
4. Neutral (neither agree nor disagree)
5. Disagree
6. Strongly disagree
7. Don’t know
8. Refuse to answer

1. If you had a child in school, you would allow him or her to be in the same classroom with another child who is infected with HIV. [STIGMAD]
2. Strongly agree
3. Agree
4. Neutral (neither agree nor disagree)
5. Disagree
6. Strongly disagree
7. Don’t know
8. Refuse to answer

1. You would not eat in a restaurant if you knew the cook was infected with HIV.

[STIGMAE]

1. Strongly agree
2. Agree
3. Neutral (neither agree nor disagree)
4. Disagree
5. Strongly disagree
6. Don’t know
7. Refuse to answer
8. You would not be willing to work next to or near a person who you knew was infected with HIV. [STIGMAF]
9. Strongly agree
10. Agree
11. Neutral (neither agree nor disagree)
12. Disagree
13. Strongly disagree
14. Don’t know
15. Refuse to answer

1. You would tell a close family member if you found out you were infected with HIV.

[STIGMAG]

1. Strongly agree
2. Agree
3. Neutral (neither agree nor disagree)
4. Disagree
5. Strongly disagree
6. Don’t know
7. Refuse to answer
8. People who have been infected with HIV should tell their new sexual partners.

[STIGMAH]

1. Strongly agree
2. Agree
3. Neutral (neither agree nor disagree)
4. Disagree
5. Strongly disagree
6. Don’t know
7. Refuse to answer

1. You would tell your current regular sexual partner (spouse, boyfriend, fiancé) if you found out you were infected with HIV. [STIGMAI]
2. Strongly agree
3. Agree
4. Neutral (neither agree nor disagree)
5. Disagree
6. Strongly disagree
7. Don’t know
8. Refuse to answer

1. It would be important to have your past sexual partners notified if you found out you were infected with HIV. [STIGMAJ]
2. Strongly agree
3. Agree
4. Neutral (neither agree nor disagree)
5. Disagree
6. Strongly disagree
7. Don’t know
8. Refuse to answer

1. Indicate whether you agree or disagree with the following statements: [Info27]
2. People can protect themselves from HIV, the virus that causes AIDS, by using a condom correctly every time they have anal sex. [KNOWLDGA]
3. Strongly agree
4. Agree
5. Neutral (neither agree nor disagree)
6. Disagree
7. Strongly disagree
8. Don’t know
9. Refuse to answer
10. Having sex with only one faithful, uninfected partner can reduce the risk of getting or giving HIV. [KNOWLDGB]
11. Strongly agree
12. Agree
13. Neutral (neither agree nor disagree)
14. Disagree
15. Strongly disagree
16. Don’t know
17. Refuse to answer
18. A healthy looking person can have HIV. [KNOWLDGC]
19. Strongly agree
20. Agree
21. Neutral (neither agree nor disagree)
22. Disagree
23. Strongly disagree
24. Don’t know
25. Refuse to answer
26. When one partner is HIV-infected and has an undetectable viral load, it is less important that a condom be used. [KNOWLDGD]
27. Strongly agree
28. Agree
29. Neutral (neither agree nor disagree)
30. Disagree
31. Strongly disagree
32. Don’t know
33. Refuse to answer
34. Because there are now good HIV medications available, I am not very concerned about getting HIV. [KNOWLDGE]
35. Strongly agree
36. Agree
37. Neutral (neither agree nor disagree)
38. Disagree
39. Strongly disagree
40. Don’t know
41. Refuse to answer
42. With receptive oral sex (sucking your partner) without a condom, there is a small but significant risk of HIV infection. [KNOWLDGF]
43. Strongly agree
44. Agree
45. Neutral (neither agree nor disagree)
46. Disagree
47. Strongly disagree
48. Don’t know
49. Refuse to answer
50. If you have anal sex initially without a condom and then put one on before ejaculating, there is a significant risk of HIV infection. [KNOWLDGG]
51. Strongly agree
52. Agree
53. Neutral (neither agree nor disagree)
54. Disagree
55. Strongly disagree
56. Don’t know
57. Refuse to answer
58. I would always have symptoms if I was infected with HIV. [KNOWLDGH]
59. Strongly agree
60. Agree
61. Neutral (neither agree nor disagree)
62. Disagree
63. Strongly disagree
64. Don’t know
65. Refuse to answer
66. I would always have symptoms if I contracted a sexually transmitted infection.

[KNOWLDGI]

1. Strongly agree
2. Agree
3. Neutral (neither agree nor disagree)
4. Disagree
5. Strongly disagree
6. Don’t know
7. Refuse to answer
8. Syphilis can be transmitted through unprotected oral sex. [KNOWLDGJ]
9. Strongly agree
10. Agree
11. Neutral (neither agree nor disagree)
12. Disagree
13. Strongly disagree
14. Don’t know
15. Refuse to answer
16. I would always have symptoms if I was infected with hepatitis C. [KNOWLDGK]
17. Strongly agree
18. Agree
19. Neutral (neither agree nor disagree)
20. Disagree
21. Strongly disagree
22. Don’t know
23. Refuse to answer
24. There is treatment currently available for hepatitis C. [KNOWLDGL]
25. Strongly agree
26. Agree
27. Neutral (neither agree nor disagree)
28. Disagree
29. Strongly disagree
30. Don’t know
31. Refuse to answer
32. I believe an HIV-infected man should be able to have a full and unlimited sex life the same as HIV-negative men. [KNOWLDGM]
33. Strongly agree
34. Agree
35. Neutral (neither agree nor disagree)
36. Disagree
37. Strongly disagree
38. Don’t know
39. Refuse to answer
40. The following questions ask about how often you have felt this way during the past week.

[Info28]

1. You felt depressed during the past week. [DEPRESSA]
2. Rarely or none of the time (less than 1 day)
3. Some or a little of the time (1-2 days)
4. Occasionally or a moderate amount of the time (3-4 days)
5. Most or all of the time (5-7 days)
6. Don’t know
7. Refuse to answer
8. You felt everything you did was an effort during the past week. [DEPRESSB]
9. Rarely or none of the time (less than 1 day)
10. Some or a little of the time (1-2 days)
11. Occasionally or a moderate amount of the time (3-4 days)
12. Most or all of the time (5-7 days)
13. Don’t know
14. Refuse to answer
15. Your sleep was restless during the past week. [DEPRESSC]
16. Rarely or none of the time (less than 1 day)
17. Some or a little of the time (1-2 days)
18. Occasionally or a moderate amount of the time (3-4 days)
19. Most or all of the time (5-7 days)
20. Don’t know
21. Refuse to answer
22. You were happy during the past week. [DEPRESSD]
23. Rarely or none of the time (less than 1 day)
24. Some or a little of the time (1-2 days)
25. Occasionally or a moderate amount of the time (3-4 days)
26. Most or all of the time (5-7 days)
27. Don’t know
28. Refuse to answer
29. You felt lonely during the past week. [DEPRESSE]
30. Rarely or none of the time (less than 1 day)
31. Some or a little of the time (1-2 days)
32. Occasionally or a moderate amount of the time (3-4 days)
33. Most or all of the time (5-7 days)
34. Don’t know
35. Refuse to answer
36. People were unfriendly during the past week. [DEPRESSF]
37. Rarely or none of the time (less than 1 day)
38. Some or a little of the time (1-2 days)
39. Occasionally or a moderate amount of the time (3-4 days)
40. Most or all of the time (5-7 days)
41. Don’t know
42. Refuse to answer
43. You enjoyed life during the past week. [DEPRESSG]
44. Rarely or none of the time (less than 1 day)
45. Some or a little of the time (1-2 days)
46. Occasionally or a moderate amount of the time (3-4 days)
47. Most or all of the time (5-7 days)
48. Don’t know
49. Refuse to answer
50. You felt sad during the past week. [DEPRESSH]
51. Rarely or none of the time (less than 1 day)
52. Some or a little of the time (1-2 days)
53. Occasionally or a moderate amount of the time (3-4 days)
54. Most or all of the time (5-7 days)
55. Don’t know
56. Refuse to answer
57. You felt that people disliked you during the past week. [DEPRESSI]
58. Rarely or none of the time (less than 1 day)
59. Some or a little of the time (1-2 days)
60. Occasionally or a moderate amount of the time (3-4 days)
61. Most or all of the time (5-7 days)
62. Don’t know
63. Refuse to answer
64. You could not get “going” during the past week. [DEPRESSJ]
65. Rarely or none of the time (less than 1 day)
66. Some or a little of the time (1-2 days)
67. Occasionally or a moderate amount of the time (3-4 days)
68. Most or all of the time (5-7 days)
69. Don’t know
70. Refuse to answer
71. Available data indicates that gay men in Toronto are still becoming infected with HIV at a high rate. Why do you think this is? [PREVNT1]

________________________________________________________________________________________________________________________________________________________________________________________________________________________

- Don’t know
- Refuse to answer

1. What more do you think we should do?  *(****click all that apply)*** PREVNT2]
2. More information and education
3. Better information
4. Greater availability of condoms
5. Better quality of condoms
6. Provide gay men at high risk for HIV the opportunity to participate in intensive prevention programs found to be effective elsewhere
7. Do more to counteract homophobia
8. Do more to counteract HIV stigma
9. Other **🡪 go to question 109.1**
10. Don’t know
11. Refuse to answer
    1. Please specify *(Use the keyboard to type or click letters on the screen)*

PREVNT3]

___________________

- Don’t know
- Refuse to answer

1. Time interview completed (Automatic variable) [ENDTIME]

*You have finished the interview. Again, thank you very much for participating today. Please call* ***Ms. Molly Gamble*** *to let her know you have finished the interview. She will come and save the information you’ve just entered.*  [Infoend]
